# hnRNPC induces isoform shifts in miR-21-5p leading to cancer development

**DOI:** 10.1038/s12276-022-00792-2

**Published:** 2022-06-21

**Authors:** Seokju Park, Hee Doo Yang, Jwa-Won Seo, Jin-Wu Nam, Suk Woo Nam

**Affiliations:** 1 Department of Life Science, College of Natural Sciences, Hanyang University, Wangsimni-ro 222, Seongdong-gu, Seoul 04763 Republic of Korea; 2 Department of Pathology, College of Medicine, The Catholic University of Korea, Banpo-daero 222, Seocho-gu, Seoul 06591 Republic of Korea; 3 Functional RNomics Research Center, The Catholic University of Korea, Banpo-daero 222, Seocho-gu, Seoul 06591 Republic of Korea; 4 Department of Information Systems, University of Maryland, Baltimore County, MD 20742 USA; 5 Research Institute for Convergence of Basic Sciences, Hanyang University, Wangsimni-ro 222, Seongdong-gu, Seoul 04763 Republic of Korea; 6 Bio-BigData Center, Hanyang Institute of Bioscience and Biotechnology, Hanyang University, Wangsimni-ro 222, Seongdong-gu, Seoul 04763 Republic of Korea; 7 Department of Biomedicine & Health Sciences, Graduate School, The Catholic University of Korea, Seoul, 06591 Korea; 8 NEORNAT Inc., The Catholic University of Korea, 222 Banpo-daero, Seocho-gu, Seoul Republic of Korea

**Keywords:** RNAi, Liver cancer

## Abstract

MicroRNA (miRNA) processing is a critical step in mature miRNA production. Its dysregulation leads to an increase in miRNA isoforms with heterogenous 5′-ends (isomiRs), which can recognize distinct target sites because of their shifted seed sequence. Although some miRNA genes display productive expression of their 5′-isomiRs in cancers, how their production is controlled and how 5′-isomiRs affect tumor progression have yet to be explored. In this study, based on integrative analyses of high-throughput sequencing data produced by our group and publicly available data, we demonstrate that primary miR-21 (pri-miR-21) is processed into the cancer-specific isomiR isomiR-21-5p | ±1, which suppresses growth hormone receptor (*GHR*) in liver cancer. Treatment with antagomirs against isomiR-21-5p | ±1 inhibited the in vitro tumorigenesis of liver cancer cells and allowed the recovery of *GHR*, whereas the introduction of isomiR-21-5p | ±1 mimics attenuated these effects. These effects were validated in a mouse model of spontaneous liver cancer. Heterogeneous nuclear ribonucleoprotein C and U2 small nuclear RNA auxiliary factor 2 were predicted to bind upstream of pre-miR-21 via a poly-(U) motif and influence Drosha processing to induce the production of isomiR-21-5p | ±1. Our findings suggest an oncogenic function for the non-canonical isomiR-21-5p | ±1 in liver cancer, and its production was shown to be regulated by hnRNPC.

## Liver cancer: Editing of microRNAs matters

Deliberate mistakes in processing of microRNAs, very short non-coding RNAs, produce isomiRs, microRNAs with altered sequences, some of which are involved in development of liver cancer. MicroRNAs regulate gene expression by binding to other genetic material based on sequence matching. IsomiRs produced by incorrect editing bind to the wrong genetic sequence and alter gene regulation networks. Jin-Wu Nam at Hanyang University and Suk Woo Nam at The Catholic University of Korea, both in Seoul, South Korea, identified two isomiRs associated with liver cancer; these isomiRs incorrectly targeted the growth hormone receptor, suppressing its expression. Treating mice with an antagonist to the isomiRs restored growth hormone receptor levels and suppressed tumor growth. These results illuminate the role of isomiRs in cancer, and may help in developing new treatments for liver cancer.

## Introduction

MicroRNAs (miRNAs) are short, ~22 nucleotide (nt), single-stranded RNAs that are involved in post-transcriptional and translational gene regulation via base pairing between their seed sequences (nt 2–7 from the 5′-end of the miRNA) and the 3′-untranslated region (3′-UTR) of their target mRNAs^1^. As seed pairing is critical for the recognition of miRNA targets, precise miRNA processing is necessary to obtain an exact seed sequence. Dysregulation of processing leads to shifts in the seed sequence and rewiring of the miRNA-target network^2–5^.

Two RNase III-type enzymes, Drosha and Dicer, are major factors that participate in the miRNA maturation process, and recognition motifs for these enzymes in primary miRNA transcripts (pri-miRNAs) and precursor miRNAs (pre-miRNAs) have been found to be critical for efficient and accurate miRNA processing^6,7^. Drosha preferentially recognizes a local hairpin structure consisting of an ~35 base pair (bp) stem between a ≥ 10-nt apical loop and a dangling single-strand region (basal segment)^8–10^. Drosha then makes a staggered cut to leave a 2-nt 3′ overhang ~13 bp away from the basal junction and/or ~22 bp away from the apical junction^8,11,12^. The basal UG and the apical UGU/GUG motif help orient the microprocessor complex on the pri-miRNA and support efficient processing^8,13,14^. The CNNC motif in the 3′ basal segment^10,13,15^ and the mismatched GHG (mGHG) motif in the basal stem^9,16^ enhance Drosha processing, and the position of these motifs affects the Drosha cleavage site. Recently, in addition to the sequence motifs, structural elements, such as the flexibility of the basal stem^17^ and the position of a bulge on the stem^10^, have also been found to modulate Drosha processing efficiency and accuracy. In contrast, the Dicer processing site is known to be determined by measuring ~22 bp from the 3′-end of the pre-miRNA (3′-counting rule)^18,19^ or from the 5′-end (5′-counting rule)^20^, or ~2 bp from the terminal/internal loop (loop-counting rule)^21^. However, both the 3′- and 5′-counting rules for Dicer processing are largely dependent on the prior Drosha processing position. After processing, miRNAs interact with Argonaute (Ago) proteins to form RNA-induced silencing complexes. Because Ago loading and target selection by the Ago-miRNA complex are also dependent on the 5′-end of the miRNA^1,22^, Drosha processing is a critical step for cognate target recognition by miRNAs in cells.

miRNA isoforms (isomiRs) with 5′-ends that differ from those of canonical miRNAs have been considered to be simple byproducts of miRNA processing, but recent reports have shown that they also have unique biological functions^2–5^. These isomiRs tend to be expressed specifically enough to distinguish between various cancer types based on their expression^23^, and some coordinate with their canonical miRNAs to regulate genes in common biological pathways^3^. For example, along with miR-140-3p, its isomiR, isomiR-140-3p, has a tumor-suppressive function in breast cancer by repressing cellular proliferation and migration^5^. However, although there are a few reports that isomiRs have functional relevance in cancer, how cancer-related isomiRs are specifically regulated and how they rewire the regulatory network to confer malignant properties on cancer cells are topics that have not yet been explored.

To comprehensively examine isomiR expression during cancer progression, we measured relative isomiR expression across various liver hepatocellular carcinoma (LIHC) datasets. While isomiR expression relative to canonical miRNA expression, henceforth referred to as the isomiR ratio, remained consistent in most cases, some isomiRs exhibited significant dysregulation in hepatocellular carcinoma (HCC) compared to non-cancerous control (non-tumor) tissues, suggesting that trans-acting factors may affect the biogenesis of isomiRs. Here, we present isomiRs of miR-21-5p (isomiR-21-5p | ±1) as potent oncomiRs that systemically inhibit the growth hormone receptor (*GHR*) and lead to the malignant transformation and growth of liver hepatocyte cells. Moreover, we demonstrate that heterogeneous nuclear ribonucleoprotein C (hnRNPC) physically interacts with primary miR-21 (pri-miR-21) to induce isomiR-21-5p | ±1, highlighting the potential therapeutic value of targeting the hnRNPC-isomiR-21-5p | ±1 regulatory axis in the treatment of liver cancer.

## Materials and methods

### Patient enrollment

Patient samples were collected at the Catholic University Hospital. The study protocol was approved by the institutional review board of the Catholic University Hospital. All investigations performed in the present study were conducted in accordance with the guidelines of the 1975 Declaration of Helsinki.

The study subjects were allocated to one of two groups: healthy controls (*n* = 15) and HCC patients (*n* = 62). Healthy individuals and patients were not randomized to conduct an observational study. Healthy controls were defined as individuals aged between 18 and 50 years. HCC was diagnosed if the tumor had a maximum diameter of >1 cm and characteristic features of HCC (arterial phase hyperenhancement, washout in the portal venous or delayed phase, threshold growth, and capsule appearance) on multiphase computed tomography and/or magnetic resonance imaging. If these criteria were present but there was a lack of diagnostic certainty, a liver biopsy was performed to confirm the diagnosis of HCC^24^. Two patient samples were excluded from the analysis because of their poor RNA-seq quality.

### Data sources

GRCh37 was used as the reference genome, GENCODE v19 (Dec-05-2013, based on GRCh37) was used for protein-coding gene annotations, and miRBase v21 (lifted over to GRCh37) was used for miRNA annotations. To acquire liver-specific 3′-UTR annotations and to measure mRNA expression levels, the 3′-UTRs were updated with the major form from profiled 3P-seq data from Huh7 cells (GSE52531)^25^. Public RNA-seq and miRNA-seq datasets from the TCGA_LIHC cohort were downloaded from the data portal of Genomic Data Commons (GDC), and those from the Tsinghua_LIHC cohort were downloaded from GSE77276^26^. To conduct pan-cancer analysis of *GHR*, gene expression and clinical information tables for 16 types of cancer were downloaded from the GDC portal, each with at least 10 nontumor and 10 cancer samples.

### qRT–PCR

Total RNA was isolated using TRIzol reagent (Invitrogen). RNA was converted to cDNA using a miScript II RT Kit (Qiagen). Reverse transcription (RT) was performed as follows: 4 µL miScript RT Reaction Buffer, 2 µL miScript RT Enzyme Mix, 2 µL miScript Nucleics Mix, and 12 µL template RNA (500 ng). The RT cycling protocol consisted of 60 min at 37 °C, 5 min at 95 °C, and cooling at 4 °C. cDNA samples were stored at -20 °C. qRT–PCR was performed using the SensiFAST SYBR No-ROX kit (Bioline), and the list of primers is shown in Supplementary Table 3. For each miRNA target, the cDNA was diluted at a ratio of 1:50. The cycling program consisted of the following: first step 10 min at 95 °C, second step (40 cycles) of denaturation (10 s at 95 °C), annealing (10 s at 58 °C), and extension (30 s at 72 °C). A Tetro cDNA synthesis kit (Bioline) was used for qRT–PCR. The RT reaction was performed as follows: 4 µL RT Reaction Buffer, 1 µL Tetro RT Enzyme, 1 µL Oligo dT, 1 µL dNTP mix, and 12 µL template RNA (1 µg). The RT cycling protocol consisted of 60 min at 37 °C, 5 min at 85 °C, and cooling at 4 °C. cDNA samples were stored at -20 °C. qRT–PCR was performed using the SensiFAST SYBR No-ROX kit (Bioline), and the list of primers is shown in Supplementary Table 4. For each mRNA target, the cDNA was diluted at a ratio of 1:20. The cycling program consisted of the first step 10 min at 95 °C and the second step cycling (40 cycles) of denaturation (10 s at 95 °C), annealing (10 s at 62 °C (GHR, GADD45G), 63.3 °C (RGS18, GPR65)), and extension (60 s at 72 °C). Raw Cq values were obtained using BioRad CFX software. The level of U6 snRNA or GAPDH expression was used as the loading control, and the relative expression levels were normalized to the control: 2^−(Target Ct − Control Ct)^.

### Cell culture and transfection

The Hep3B, HepG2, Huh7, PLC/PRF/5, SNU-182, SNU-354, SNU-368, SNU-387, SNU-423, SNU-449, and SNU-475 HCC cell lines were acquired from the Korean Cell Line Bank. The normal liver cell line MIHA was kindly provided by Dr. Roy-Chowdhury (Albert Einstein College of Medicine). All cell lines were maintained in RPMI-1640, DMEM, or EMEM supplemented with 10% fetal bovine serum and 100 units/mL penicillin/streptomycin (GenDepot). All cells were cultured at 37 °C in a humidified incubator with 5% CO_2_.

Small interfering RNAs (siRNAs) were synthesized by Genolution (Seoul, Korea), antisense antagomirs were purchased from BIONEER (Daejeon, Korea), and 100 nM siRNA was transfected into liver cancer cells. The siRNA or antagomir sequences are listed in Supplementary Table 4. Human hnRNPC and U2AF2 expression plasmids and subcloned gene ORF sequences were purchased from GenScriptTM (Piscataway, NJ, USA). Transfections were carried out using Lipofectamine RNAiMAX or Lipofectamine 2000 reagent (Invitrogen) according to the manufacturer’s instructions.

### Quantification and statistical analysis

DEG analysis between two groups was performed using the Wilcoxon rank-sum test for unpaired data and the Wilcoxon signed rank-sum test for paired data with the *mannwhitneyu* function in the Python Scipy package. Pearson correlation coefficients were calculated using the *Pearsonr* function in the Python Scipy package. Univariate and multivariate analyses of survival differences between the two patient groups, divided by the median expression level, were conducted using the survival package in R. Cox proportional hazard ratios were calculated using the *coxph* function, and *P* values were estimated using the Wald test. Multivariate linear regression was performed using the R built-in function ′lm′ after min-max scaling of all variables to facilitate the comparison of coefficients. Multiple hypothesis correction was performed using Benjamini–Hochberg FDR correction. All quantitative experiments were reported with data from three independent biological replicates, and the results are presented as the mean ± SEM. Statistical comparisons between two groups were performed using an unpaired Student’s T test.

## Results

### IsomiR expression in non-tumor hepatocytes and HCCs

**Fig. 1:**
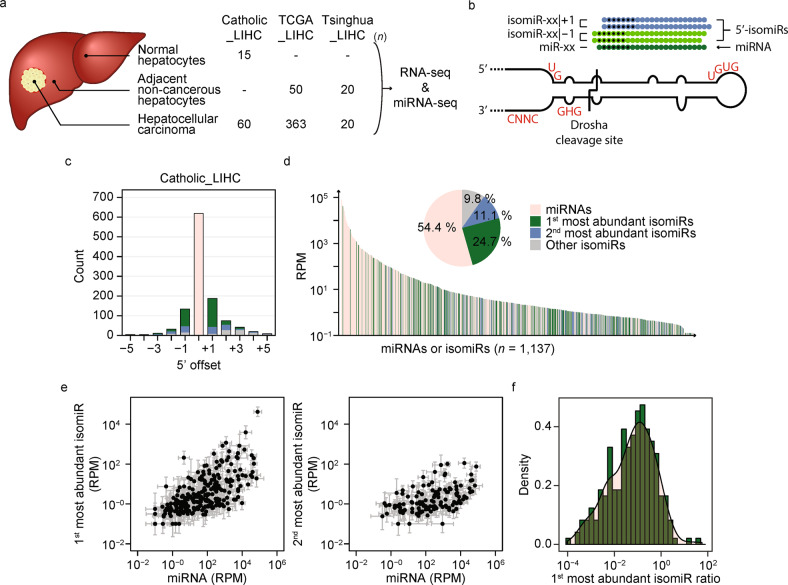
IsomiR expression in non-tumor hepatocytes and LIHCs. **a** A cartoon that depicts the sample preparation sites in the liver together with a table summarizing the number of samples for which RNA-seq and miRNA-seq were performed in each cohort. **b** A cartoon that depicts the structure of a pri-miRNA with previously studied motifs that regulate the Drosha cleavage site, including the basal UG, apical UGU/GUG, flanking CNNC, and mismatched GHG motifs. Mature miRNAs are classified as miRNAs or isomiRs and are named according to their 5′-end. Black dots indicate the seed regions. **c** The number of expressed miRNAs and isomiRs in the Catholic_LIHC. **d** The pool of expressed miRNAs/isomiRs ordered by their expression level in the Catholic_LIHC. The x-axis indicates the individual miRNAs/isomiRs, and the y-axis indicates the median RPM for all samples. The inset shows the relative abundance of miRNAs and isomiRs. **e** Comparison of the differences in expression levels between miRNAs and the most abundant isomiRs (left) and between miRNAs and the second most abundant isomiRs (right). Data are represented as the median ± SD**. f** Density plot of the means of isomiR ratios for the most abundant isomiR for all samples.

To identify cancer-specific isomiR signatures, we initially processed our multi-stage HCC data (Catholic_LIHC) and publicly available liver cancer miRNA sequencing datasets obtained from The Cancer Genome Atlas liver HCC project (TCGA_LIHC) and the Gene Expression Omnibus (GEO) of the National Center for Biotechnology Information (NCBI) (GSE77276, Tsinghua_LIHC) (Fig. 1a). Data were mapped to hairpin precursor sequences in miRBase (version 21) using miRDeep2^27^, which was subjected to our computational pipeline (Fig. 1b and Supplementary Table 1; see Methods for more details). Because differences in the 5′- but not 3′-ends of the isomiRs shifted seed sequences and altered target mRNAs, mapped reads with 3′-end offsets were collapsed to the same miRNAs/isomiRs (Fig. 1b). Eighteen miRNAs that were found to be dominantly processed at their 5′-ends differently than previously annotated in miRBase were accordingly updated with the new 5′-end, and the original miRNAs were treated as isomiRs (Supplementary Fig. 1a and Supplementary Table 2). Collectively, 619 miRNAs and 518 isomiRs (from 466 miRNA loci) with ≥ 1 reads per million mapped reads (RPM) were detected in at least seven samples from Catholic_LIHC cohorts. The majority of the isomiR 5′-ends were offset ± by 1 nt from the canonical 5′-ends (Fig. 1c), whereas 18% of the isomiRs had 5′-ends that were offset by more than ±1 nt. IsomiRs accounted for approximately 45% of all detected miRNAs (Fig. 1d, inset), and 10 isomiRs were ranked within the top 100 most abundant miRNAs (Fig. 1d), suggesting that isomiRs should be taken into account in miRNA studies, as they could play roles that are just as pivotal as those of canonical miRNAs in pathogenic conditions such as cancer.

IsomiR expression levels tended to be highly correlated with those of the corresponding miRNAs in general, as they were processed from the same pri-miRNAs (Supplementary Fig. 1b). However, some miRNA genes appeared to generate more isomiRs than others, even though the corresponding miRNAs were expressed at similar levels (Fig. 1e). Additional analyses showed that the isomiR ratio varied by miRNA in all tested cohorts (Fig. 1f and Supplementary Fig. 1c–f).

### Identification and characterization of liver cancer-specific isomiRs

Given these observations, we hypothesized that the isomiR level could be attributed not only to the pri-miRNA level but also to the isomiR production rate. To understand how the isomiR production rate affects the isomiR level, the normalized reads per million (RPM) numbers of isomiRs were regressed with those of miRNAs and the isomiR ratios using multivariate linear regression. The isomiR ratio was significantly correlated with the isomiR level (Supplementary Fig. 2a; coefficient = 0.037; coefficient = 0.014; coefficient = 0.065 in Catholic_LIHC, TCGA_LIHC, and Tsinghua_LIHC, respectively; *P* < 2 **×** 10^−16^ in all cohorts). We examined (possible) sources of isomiR ratio variation, and it appeared that the isomiR ratios were highly variable across miRNA genes but remained mostly stable across different cohorts (Supplementary Fig. 2b; *r* ≥ 0.91), indicating that the isomiR production rate is mostly determined by intrinsic factors (sequence and structure) of miRNA genes, as previously reported for in vitro studies^9,16^. For example, the mir-122 gene produced few isomiRs (< 0.1%) in all three cohorts, whereas the mir-192 gene generated abundant isomiRs (29–41%) (Supplementary Fig. 2b, c).

**Fig. 2:**
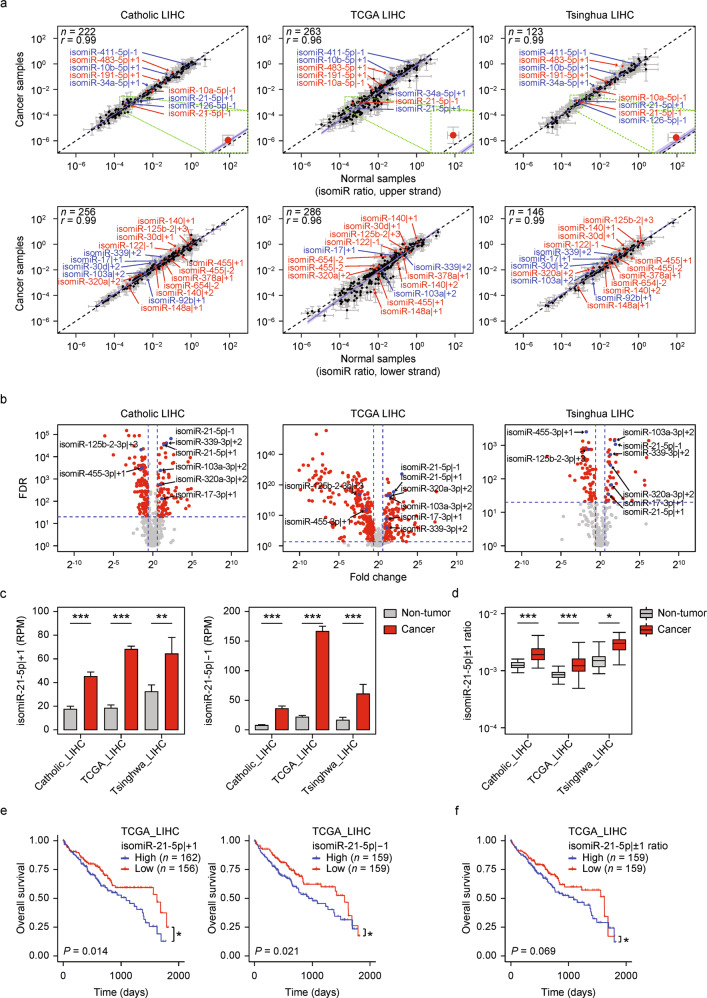
Identification and characterization of liver cancer-specific isomiR-21-5p | ±1. **a** Comparison of isomiR ratios between non-tumor and HCC samples in each cohort. Red and blue dots indicate isomiRs that exhibit significantly changed isomiR ratios (FDR ≤ 0.05) in all cohorts and in both Catholic_LIHC and Tsinghua_LIHC, respectively. The top plots show miRNAs/isomiRs that were generated from the upper strand of the pri-miRNA, and the bottom plots show those generated from the lower strand. The purple line and light-purple shade along the purple line represent the corresponding linear regression line and 95% confidence intervals for the slope of the regression line, respectively. Gray whiskers indicate standard deviations between samples. The insets highlighted in green show the isomiR-21-5p | ±1 ratio as an example. **b** Volcano plots of differential isomiR expression between non-tumor and HCC samples. IsomiRs that were significantly changed in HCCs are indicated by blue dots. **c** Comparison of the expression levels of isomiR-21-5p | ±1 between non-tumor and HCC samples in each cohort. **d** Difference between the isomiR-21-5p | ±1 ratio in non-tumor and HCC samples in each cohort. Boxes represent quantile distributions of isomiR-21-5p | ±1 ratios, and whiskers extend up to 1.5 x IQR (interquantile range). **e** Kaplan–Meier plot showing the overall survival stratified by the expression of isomiR-21-5p | +1 (left) and isomiR-21-5p | -1 (right) from the TCGA_LIHC. **f** Kaplan–Meier plot showing the overall survival stratified by the isomiR-21-5p | ±1 ratio from the TCGA_LIHC. Data represent the median in **b** and the median ± SD in **a,** **c**. Statistical significance was determined by one-way ANOVA test **a**, one-tailed Wilcoxon rank-sum test **b**–**d** with the exception of the Tsinghua_LIHC, for which the one-tailed Wilcoxon signed rank-sum test was used, and Wald test **e** and **f**, *FDR ≤ 0.05, **FDR ≤ 0.01, and ***FDR ≤ 0.001.

We then determined the specific isomiRs that were differentially regulated in liver cancer. We compared the ratios of the first and second most abundant isomiRs derived from a given miRNA in non-tumor and HCC samples and found that the ratios were mostly constant (Fig. 2a). This finding indicates that the production rate of most isomiRs is maintained in HCCs. However, the ratios of 25 isomiRs (~10% of commonly profiled isomiRs from the miRNA-seq datasets) were repeatedly found to differ between non-tumor and HCC samples in all cohorts (Fig. 2a), suggesting context-dependent regulation of isomiR production in HCCs. For instance, mir-21 produced relatively more isomiR-21-5p | +1 and isomiR-21-5p | -1 in tumor tissues than in non-tumor tissues (false discovery rate, FDR, < 3.74 × 10^−4^; FDR < 5.61 × 10^−8^, respectively; one-way ANOVA test). Taken together, these results indicate that although isomiR production rates seem to be dominantly regulated by the cis-acting elements of miRNAs, some can be modulated by cellular context-dependent factors, such as trans-acting elements.

### Clinically relevant isomiR-21-5p | ±1

We further prioritized the isomiRs by inspecting differentially expressed genes (DEGs) and their association with clinical information (Supplementary Fig. 7a). Of the eight isomiRs whose levels appeared to be significantly changed in HCC across cohorts (Fig. 2b), three isomiRs (isomiR-103a-3p | +2, isomiR-21-5p | +1, and isomiR-21-5p | -1) were associated with overall patient survival rates (*P* ≤ 0.05, for at least one dataset, Wald test) (Supplementary Fig. 2d). The expression levels of isomiR-103a-3p | +2 were relatively low (Supplementary Fig. 2e) compared to those of both isomiR-21-5p | ±1, which exhibited significant overexpression and an increase in the isomiR ratio in HCC (Fig. 2c, d). Both isomiR-21-5p | ±1 levels were significantly associated with the overall survival rates (Fig. 2e; *P* ≤ 0.05, Wald test). High miR-21-5p expression was also associated with a poor survival rate in patients with liver cancer, implying that the clinical relevance of isomiR-21-5p | ±1 may come from that of miR-21-5p (Supplementary Fig. 2f). To partly overcome the collinearity problem between the expression of isomiR-21-5p | ±1 and miR-21-5p (Supplementary Fig. 2g), survival analysis was performed using the isomiR ratio rather than the expression levels. Patients with liver cancer with high isomiR ratios exhibited marginally worse survival rates, indicating that isomiR-21-5p | ±1 and miR-21-5p had independent clinical relevance (Fig. 2f).

### IsomiR-21-5p | ±1 function as an oncomiR in liver cancer

**Fig. 3:**
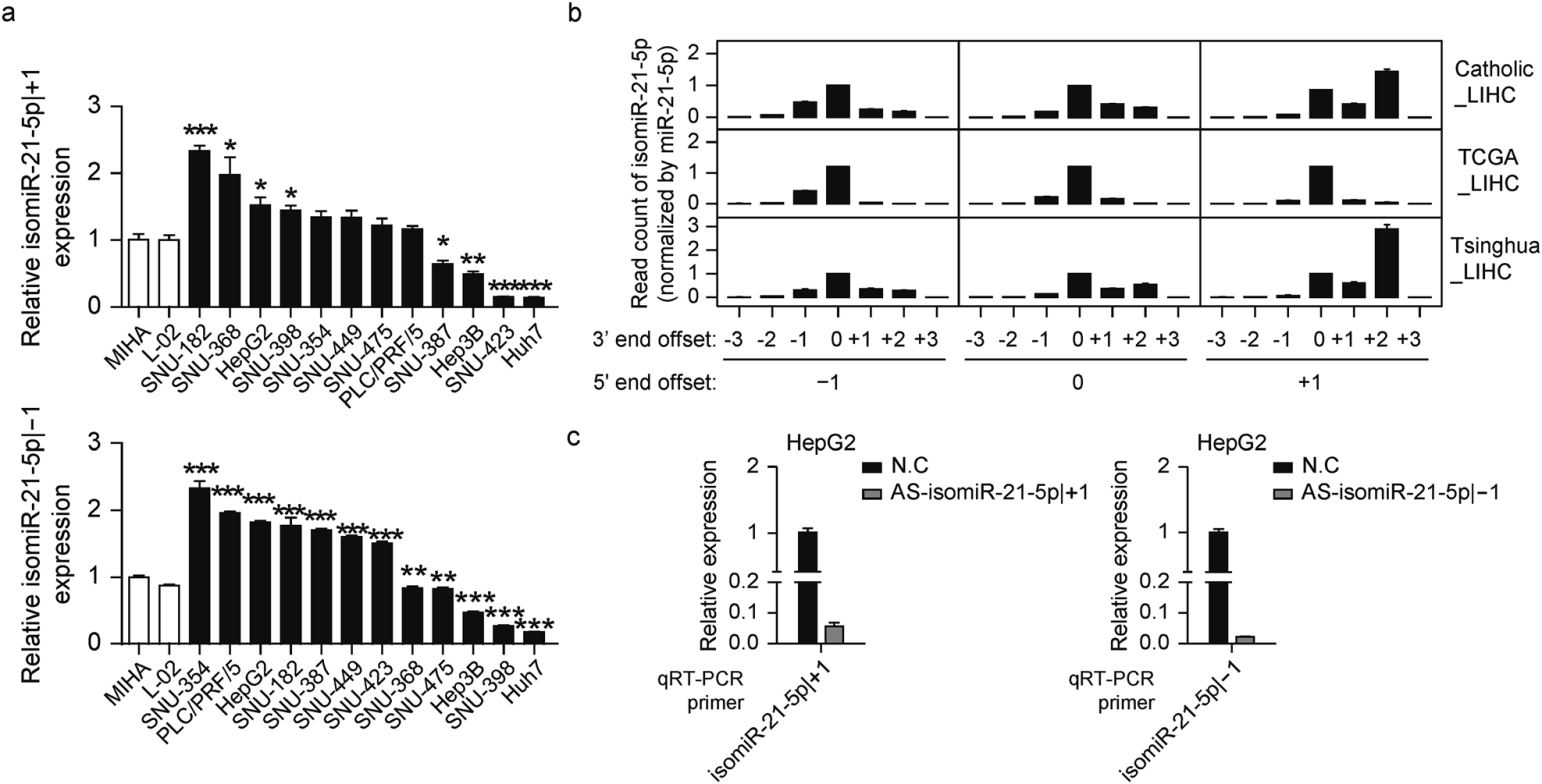
Validation of isomiR-21-5p | ±1 quantification and perturbation methods. **a** qRT–PCR was performed to quantify the level of isomiR-21-5p | +1, top plot, and isomiR-21-5p | -1, bottom plot, in two normal liver cell lines, L-02 and MIHA, and 12 liver cancer cell lines, Hep3G, HepG2, Huh7, PLC/PRF/5, SNU-182, SNU-354, SNU-368, SNU-387, SNU-398, SNU-423, SNU-449, and SNU-475. **b** The miRNA read counts of miR-21-5p and its 5′-isomiRs, isomiR-21-5p | +1 and isomiR-21-5p | -1, shown for each 3′-end offset, profiled from the Catholic_LIHC, TCGA_LIHC, and Tsinghua_LIHC cohorts. **c** HepG2 cells were transfected with AS-isomiR-21-5p | +1, left plot, or AS-isomiR-21-5p | -1, right plot, after which qRT–PCR was performed to quantify the levels of isomiR-21-5p | +1 or isomiR-21-5p | -1, respectively. Data represent the median ± SD in **b** and the median ± SEM of three independent experiments/three replicates in **a**, **c**. Statistical significance was determined by Student’s t test **a**, ns: no significance, *FDR ≤ 0.05, **FDR ≤ 0.01 and ***FDR ≤ 0.001.

We assessed the endogenous expression levels of isomiR-21-5p | ±1 in liver cancer cell lines and immortalized normal hepatocytes (MIHA and L-02). Two liver cancer lines, SNU-182 and SNU-368, exhibited relatively high expression of isomiR-21-5p | +1 compared to MIHA and L-02 (Fig. 3a, top), whereas the liver cancer lines SNU-354 and PLC/PRF/5 exhibited high isomiR-21-5p | -1 expression (Fig. 3a, bottom). Next, we introduced mimics of isomiR-21-5p | ±1 into liver cancer cells expressing relatively low levels of isomiR-21-5p | ±1 [Hep3B and Huh7 (both mimics individually introduced), SNU-423 (isomiR-21-5p | +1 mimic), and SNU-398 (isomiR-21-5p | -1 mimic)]. The expression of each isomiR-21-5p | ±1 was successfully measured in liver cancer cells and was distinguished from that of miR-21-5p (Supplementary Fig. 3a, b).

Interestingly, we found that isomiR-21-5p | ±1 is produced with different 3′-ends in a manner that is partly dependent on the 5′-end (Fig. 3b), which was previously reported as the 5′-counting rule of Dicer^20^. Therefore, to reduce possible experimental bias, we designed primers for the major 3′-ends to determine isomiR expression levels. We examined whether isomiR-21-5p | ±1 was downregulated by antisense isomiR-21-5p | ±1 (AS-isomiR-21-5p | -1 and AS-isomiR-21-5p | +1). Treatment with either form of antisense led to targeted disruption of the indicated versions of isomiR-21-5p | ±1 in HepG2 liver cancer cells, which normally express both isomiR-21-5p | ±1 at high levels (Fig. 3c). Similarly, treatment with AS-isomiR-21-5p | +1 reduced the levels of endogenous isomiR-21-5p | +1 in SNU-182 and SNU-368 cells, which normally express isomiR-21-5p | +1 at high levels, whereas treatment with AS-isomiR-21-5p | -1 reduced the levels of endogenous isomiR-21-5p | -1 in PLC/PRF/5 and SNU-354 cells, which normally express isomiR-21-5p | -1 at high levels (Supplementary Fig. 3c, d). Therefore, we assessed the biological role of isomiR-21-5p | ±1 in the development and progression of liver cancer using these antisense constructs.

**Fig. 4:**
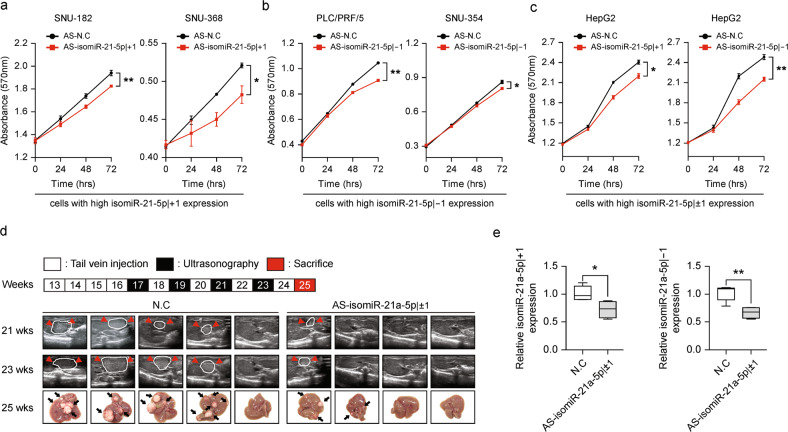
IsomiR-21-5p | ±1 function as an oncomiR in liver cancer. **a** SNU-182 (left panel) and SNU-368 (right panel) cells were transfected with AS-negative control or AS-isomiR-21-5p | +1, and MTT assays were performed to determine the effect on cell proliferation. **b** PLC/PRF/5 (left panel) and SNU-354 (right panel) cells were transfected with AS-negative control or AS-isomiR-21-5p | -1, and MTT assays were performed to determine the effect on cell proliferation. **c** HepG2 cells were transfected with AS-negative control or AS-isomiR-21-5p | +1, left panel, or AS-negative control or AS-isomiR-21-5p | -1, right panel, and MTT assays were performed to determine the effect on cell proliferation. **d**, **e** Tumor growth in a mouse model of liver cancer following tail vein injection of AS-isomiR-21-5p | ±1. **d** (Top) Schematic flow of the experiment, showing the timing of tail vein injection, ultrasonography, and sacrifice. (Bottom) Pictures showing ultrasonography results at 21 wks and 23 wks and the livers from mice sacrificed at 25 wks. **e** qRT–PCR was performed to quantify isomiR-21a-5p | +1, left panel, and isomiR-21a-5p | -1, right panel, in the livers from sacrificed mice. U6 was used as a loading control. Data in **a**–**c** are represented as the mean ± SEM; Student’s t test, *P ≤ 0.05, **P ≤ 0.01, and ***P ≤ 0.001.

To verify the oncogenic function of isomiR-21-5p±1 in liver carcinogenesis, we performed an MTT assay. The introduction of AS-isomiR-21-5p | +1 into cells that highly expressed isomiR-21-5p | +1 (SNU-182 and SNU-368) significantly suppressed tumor cell growth rates (Fig. 4a). Similarly, treatment of cells that highly express isomiR-21-5p | -1 (PLC/PRF/5 and SNU-354) with AS-isomiR-21-5p | -1 led to an anti-growth effect, similar to the treatment of HepG2 cells that express both isomiR-21-5p | ±1 at high levels, with either AS-isomiR-21-5p | +1 or AS-isomiR-21-5p | -1 (Fig. 4b, c).

Next, to validate our in vivo observations, *Ras*-Tg mice that spontaneously developed HCC at approximately 15 weeks of age were established^28^. Murine mir-21a expressed both isomiR-21a-5p | +1 and isomiR-21a-5p | -1 in liver tissue (Supplementary Fig. 4a). Mice were injected with AS-isomiR-21a-5p | ±1 intravenously with the liver-specific delivery reagent Invivofectamine weekly starting at 13 weeks of age. Liver cancer was detected in the mice using ultrasonography starting at 17 weeks of age (Fig. 4d). Liver cancer masses were detectable starting at 17 weeks of age in the negative control group (N.C), in which four of the five mice developed multiple, large liver cancers. However, liver cancer occurred in only two of the four mice, in which isomiR-21-5p | ±1 levels were reduced (Supplementary Fig. 4b, c). Suppression of isomiR-21a-5p |±1 was observed in the groups treated with AS-isomiR-21a-5p | ±1 (Fig. 4e).

### Aberrantly upregulated hnRNPC induces isomiR-21-5p | ±1 in liver cancer

**Fig. 5:**
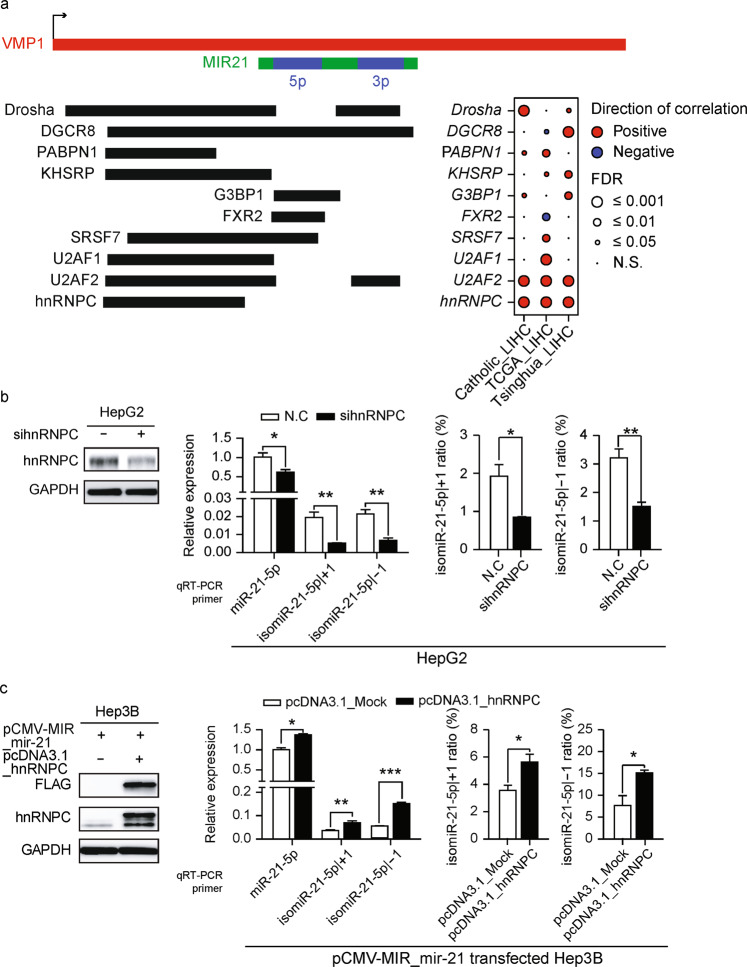
Aberrantly upregulated hnRNPC induces isomiR-21-5p | ±1 in liver cancer. **a** The schematic illustrates a pre-miR-21 region, its host gene, VMP1, and RBPs with their binding sites in the flanking region of pre-miR-21. The inset shows Pearson correlations between the isomiR-21-5p | ±1 ratio and the expression of bound RBPs in each cohort. **b** HepG2 cells were transfected with sihnRNPC. qRT–PCR was conducted to quantify the levels of miR-21-5p, isomiR-21-5p | +1, and isomiR-21-5p | -1, after which the isomiR ratios were calculated. **c** Hep3B cells were transfected with pcDNA3.1_hnRNPC in the pCMV-MIR-mir-21-transfected background. qRT–PCR was conducted to quantify the levels of miR-21-5p, isomiR-21-5p | +1, and isomiR-21-5p | -1, after which the isomiR ratios were calculated. Data represent the mean ± SEM of three independent experiments/three replicates **b,** **c**. Statistical significance was determined by Student’s t test **b**, **c**, ns: no significance, *FDR ≤ 0.05, **FDR ≤ 0.01, and ***FDR ≤ 0.001.

Previous studies have reported that several sequence and structural motifs, such as CNNC, mGHG, basal UG, and basal junction, affect the specificity of Drosha cleavage sites as well as miRNA processing efficiency^9,15,16^. As expected, we found that most of these motifs were significantly associated with isomiR ratios (Supplementary Fig. 5a–d). However, as the cis-acting elements were neither mutated nor edited in our HCC samples (data not shown), we suspected the involvement of trans-acting elements, such as RNA-binding proteins (RBPs), which can affect the production of isomiRs depending on the pathological condition. RBP binding to the pri-miRNA could alter the secondary structure of the pri-miRNA or destabilize microprocessor binding, possibly resulting in the alteration of Drosha cleavage sites. Notably, an analysis of RBPs in the HepG2 liver cancer cell line using eCLIP-seq data^29^ identified 10 RBPs that bind to the precursor miR-21 (pre-miR-21-5p) and its flanking region (Fig. 5a). The levels of *U2AF2* and *hnRNPC* appeared to be significantly correlated with the isomiR ratio in all the tested cohorts (FDR < 0.001). To exclude the possibility that the association was due to other factors, such as the level of the host transcript, VMP1, or miR-21-5p, we performed multivariate analysis. The levels of *U2AF2* and *hnRNPC* exhibited a significant relationship with the isomiR-21-5p | ±1 ratio (*P* < 0.01; Supplementary Fig. 5e, f). Moreover, both RBPs were consistently overexpressed in the tested cohorts, except for *U2AF2* in Catholic_LIHC (Supplementary Fig. 5g). Therefore, we hypothesized that hnRNPC and/or U2AF2 binding to pri-miR-21 modulates the Drosha cleavage sites in pri-miR-21 to produce more isomiR-21-5p | ±1 in liver cancer.

To validate this hypothesis, we used RNA interference to knockdown hnRNPC and U2AF2 expression in liver cancer cells, after which miR-21-5p and isomiR-21-5p | ±1 expression levels were measured. Both the isomiR-21-5p | ±1 level and isomiR ratio were significantly reduced in both hnRNPC and U2AF2 knockdown cells compared to controls in all tested liver cancer cell lines (Fig. 5b and Supplementary Fig. 5h, i, m–o). To better understand these results, an hnRNPC overexpression construct was introduced into liver cancer cells along with a pri-miR-21-expressing plasmid, after which changes in miR-21-5p and isomiR-21-5p | ±1 levels were examined. As expected, hnRNPC overexpression caused an increase in both isomiR-21-5p | ±1 expression and the isomiR ratio in all tested liver cancer cell lines (Fig. 5c and Supplementary Fig. 5j–l). The results were similar in U2AF2- and hnRNPC-overexpressing cells, but slightly less significant changes were associated with U2AF2 overexpression (Supplementary Fig. 5p–s). These results suggest that hnRNPC plays an important role in the processing of isomiR-21-5p | ±1 in liver carcinogenesis.

### An hnRNPC-isomiR-21-5p | ±1 regulatory axis contributes to liver carcinogenesis

**Fig. 6:**
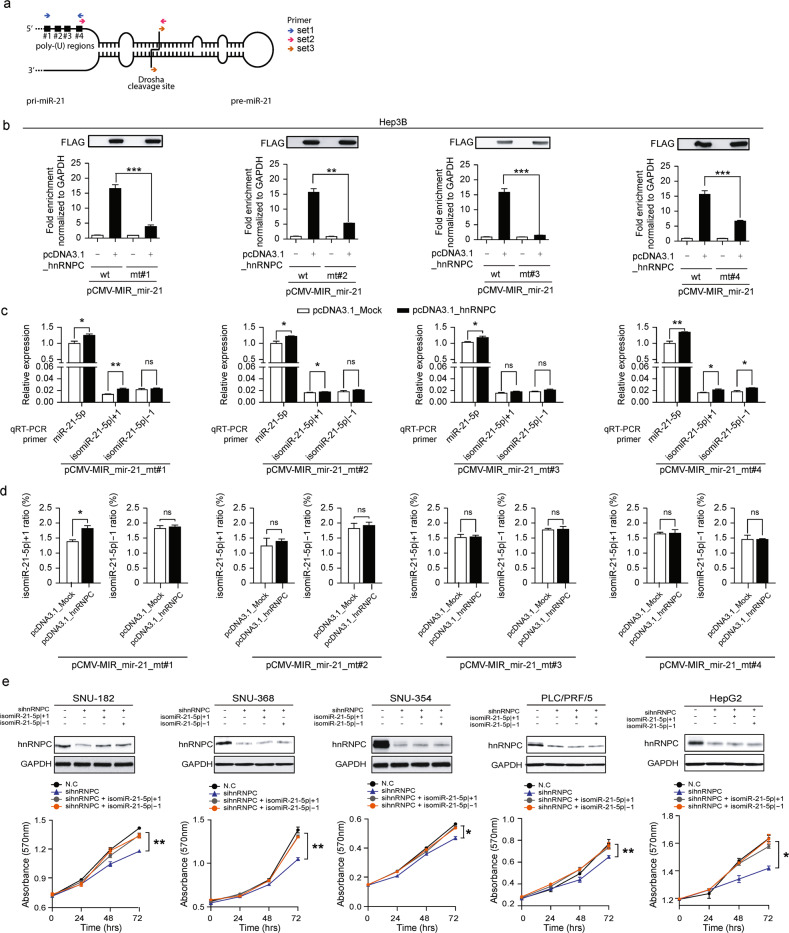
hnRNPC-isomiR-21-5p | ±1 regulatory axis contributes to liver carcinogenesis. **a** A cartoon of the pri-miR-21 structure with four hnRNPC binding motifs. The primer sets used for determining the levels of pri-miR-21 are indicated. **b** Immunoprecipitation of FLAG-tagged hnRNPC was performed to investigate hnRNPC binding affinity to pri-miR-21. The levels of pri-miR-21 in the immunoprecipitated materials were determined by qRT–PCR using primer set 3. Hep3B cells were transfected with pCMV-MIR_mir-21, encoding either wt pri-miR-21 or versions with mutations in each of the hnRNPC binding motifs (mt#1–4), and pcDNA3.1_hnRNPC. **c**, **d** qRT–PCR was performed to measure the expression levels of the isomiRs (**c**), after which the isomiR ratios were calculated (**d**). Hep3B cells were transfected as in Fig. 6b. **e** SNU-182, SNU-368, SNU-354, PLC/PRF/5, and HepG2 cells were transfected with sihnRNPC, isomiR-21-5p | +1, or isomiR-21-5p | -1. The levels of hnRNPC and GAPDH expression were analyzed by Western blotting (upper panel), and cell growth was analyzed using MTT assays (lower panel). Data represent the mean ± SEM of three independent experiments/three replicates **b**–**e**. Statistical significance was determined by Student’s t test **b**–**e**, ns: no significance, *FDR ≤ 0.05, **FDR ≤ 0.01, and ***FDR ≤ 0.001.

In general, hnRNPC binds to a wide region of RNA that is approximately 230 nt in length^30–32^. Therefore, to validate the regulatory roles of hnRNPC in inducing isomiR-21-5p | ±1 in liver carcinogenesis, we constructed pri-miR-21 expression plasmids containing random mutations in four different poly-(U) regions of pri-miR-21, known as hnRNPC binding motifs (Fig. 6a). Hep3B cells, which exhibited relatively low expression of both endogenous isomiR-21-5p | ±1, were transfected with wild-type (wt) pri-miR-21- or mutated pri-miR-21 (mt#1–4)-expressing plasmids in the presence or absence of FLAG-tagged hnRNPC overexpression. First, we performed hnRNPC immunoprecipitation in cells transfected with wt pri-miR-21-expressing plasmids and determined the levels of pri-miR-21 and/or pre-miR-21 in the immunoprecipitated materials using three different sets of primers (Fig. 6a and Supplementary Fig. 6a). As expected from the eCLIP-seq results (Fig. 5a), hnRNPC bound to pri-miR-21 rather than pre-miR-21. Immunoprecipitation was also performed in cells transfected with mutated pri-miR-21-expressing plasmids, and the results showed that all mutations attenuated hnRNPC binding to pri-miR-21 (Fig. 6b). In addition, the same mutant-transfected cells exhibited reduced amounts of both isomiR-21-5p | ±1 compared to the wt-transfected cells (Figs. 5c, 6c). Moreover, the isomiR ratios for the same cells showed no significant changes compared with those in the corresponding control, with the exception of the isomiR-21-5p | +1 ratio in cells expressing the mt#1 construct (Fig. 6d).

To validate the existence of an hnRNPC-isomiR-21-5p | ±1 regulatory axis in liver carcinogenesis, liver cancer cells were treated with short interfering RNAs targeting hnRNPC (sihnRNPCs) followed by treatment with isomiR-21-5p | ±1 mimics. As shown in Fig. 6e, the anti-growth effect of hnRNPC inhibition was mostly rescued by the introduction of isomiR-21-5p | ±1 mimics in all the tested liver cancer cell lines. These results indicate that aberrant upregulation of hnRNPC induces isomiR-21-5p | ±1 during liver carcinogenesis and contributes to the malignant transformation and growth of non-cancerous hepatocytes.

### Cancer-related targets of isomiR-21-5p | ±1

Of the 13,143 mRNAs commonly profiled from the RNA-seq datasets, 3,049 cancer-related mRNAs were selected using gene ontology (GO) analysis (Supplementary Fig. 7a). We further prioritized the genes by inspecting differentially expressed genes (DEGs) and their association with clinical information. Of the 534 mRNAs whose levels appeared to be significantly changed in HCC across cohorts, 187 mRNAs were associated with overall patient survival rates (*P* ≤ 0.05, for at least one dataset, Wald test).

**Fig. 7:**
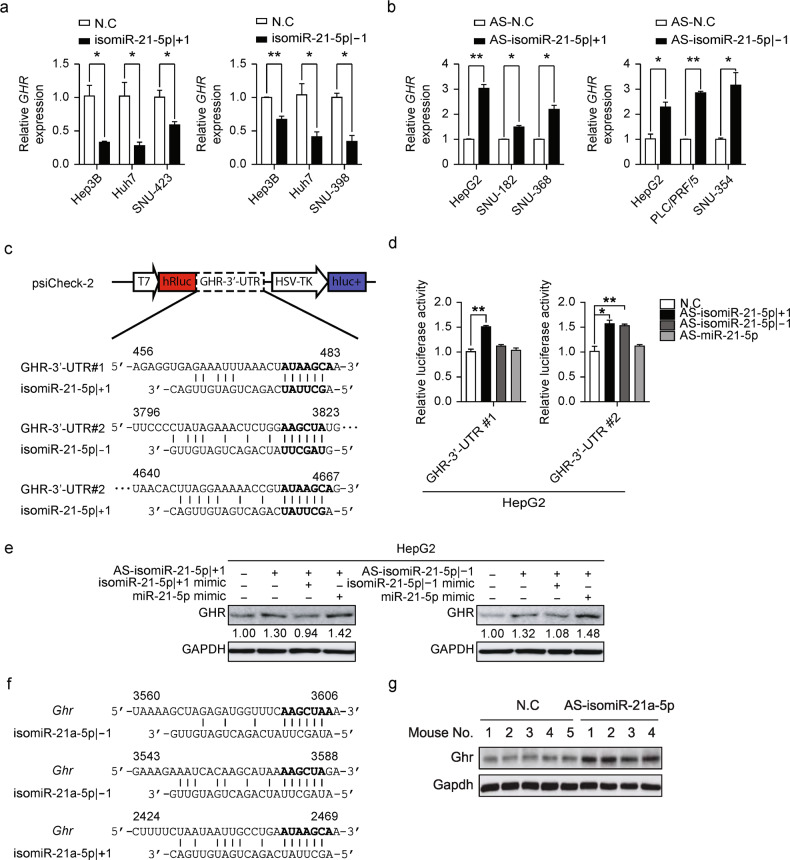
Clinically relevant target of isomiR-21-5p | ±1. **a** Hep3B, Huh7, and SNU-423 cells were transfected with the isomiR-21-5p | +1 mimic, left panel, or Hep3B, Huh7, and SNU-398 were transfected with the isomiR-21-5p | -1 mimic, right panel. qRT–PCR was performed to quantify the level of *GHR*. **b** HepG2, SNU-182, and SNU-368 cells were transfected with AS-isomiR-21-5p | +1, left panel, or HepG2, PLC/PRF/5, and SNU-354 were transfected with AS-isomiR-21-5p | -1, right panel. Otherwise, as in Fig. 7a. **c** Schematic representation of vector constructions including the predicted binding sites of isomiR-21-5p | ±1 in the 3′-UTR of *GHR*. **d** HepG2 cells were cotransfected with AS-isomiR-21-5p | +1, AS-isomiR-21-5p | -1, AS-miR-21-5p, or control, and one of two psiCHECK-2-GHR-3′-UTR vectors (*GHR* 3′-UTR Renilla luciferase reporters that also encode firefly luciferase). Both Renilla and firefly luciferase activities were measured in the same sample. Renilla luciferase signals were normalized to firefly luciferase levels. **e** HepG2 cells were transiently transfected with a combination of AS-isomiR-21-5p | +1, isomiR-21-5p | +1, and miR-21-5p, left panel, or AS-isomiR-21-5p | -1, isomiR-21-5p | -1, and miR-21-5p, right panel. Western blotting was performed to detect GHR and GAPDH. The relative density of each band was analyzed using ImageJ. **f** A cartoon showing the predicted target sites of isomiR-21a-5p | ±1 in the 3′-UTR of *Ghr*. **g** Western blotting was performed to quantify Ghr and Gapdh levels in the livers from the mice sacrificed, as shown in Fig. 4d. Data in **a**, **b**, and **d** are represented as the mean ± SEM; Student’s t test, *P ≤ 0.05 and **P ≤ 0.01.

We then predicted target genes regulated by isomiR-21-5p | ±1 in liver cancer using TargetScan v7.0^33^. Of the 187 mRNAs, 14 were identified as putative targets of isomiR-21-5p | ±1 with at least one target site (6-mer sites with less than -0.1 weighted context ++ score or other canonical site types^34–37^) (Supplementary Fig. 7b). Because both isomiR-21-5p | ±1 displayed positive effects on cell growth, of the clinically relevant isomiR-21-5p | ±1 targets, we focused on *RGS18*, *GADD45G*, *GPR65*, and *GHR*, which contain target sites for both isomiR-21-5p | +1 and isomiR-21-5p | -1 in the 3′-UTR (Supplementary Fig. 7c). First, the introduction of isomiR-21-5p | +1 or isomiR-21-5p | -1 mimics in cells with low isomiR-21-5p | ±1 expression consistently suppressed *GHR* expression, whereas treatment with AS-isomiR-21-5p | +1 or AS-isomiR-21-5p | -1 significantly induced *GHR* expression in cells with high isomiR-21-5p |±1 expression (Fig. 7a, b). These manipulations led to varied results for *RGS18*, *GADD45G*, and *GPR65* in the same experiments (Supplementary Fig. 7d–i). To validate the specificity and selectivity of isomiR-21-5p | +1 and isomiR-21-5p | -1 targeting *GHR*, we constructed two different psiCHECK-2-GHR-3′-UTR vectors containing either the isomiR-21-5p | +1 site (*GHR*-3′-UTR#1) or both isomiR-21-5p | ±1 sites (*GHR*-3′-UTR#2) downstream of a luciferase reporter gene (Fig. 7c) and transformed them into liver cancer cells. As expected, treatment with AS-isomiR-21-5p | ±1, but not with AS-miR-21-5p, significantly augmented luciferase activity (relative to the activity of a second luciferase expressed from the vector) in an isomiR target sequence-specific manner in all tested liver cancer cells (Fig. 7d and Supplementary Fig. 7j, k). The selective regulation of *GHR* by isomiR-21-5p | ±1 was further validated by Western blot analysis (Fig. 7e). GHR expression was suppressed by the introduction of AS-isomiR-21-5p | +1 or AS-isomiR-21-5p | -1 into HepG2 cells, in which both isomiR-21-5p | ±1 were highly expressed and selectively suppressed by treatment with mimics of either isomiR-21-5p | +1 or isomiR-21-5p | -1 but not by treatment with miR-21-5p. Moreover, murine *Ghr* also includes target sites for isomiR-21a-5p | ±1 but not for miR-21a-5p, similar to the situation in humans (Fig. 7f). Western blot analysis confirmed elevated Ghrs expression in mouse liver tissues in the groups treated with AS-isomiR-21a-5p | ±1 (Figs. 4d, 7g).

## Discussion

IsomiRs, characterized by variation at the 3′- and/or 5′-end(s) of canonical miRNAs, can be created either by imprecise processing by Drosha or Dicer or through the addition or removal of nucleotides at the 3′-end during miRNA biogenesis^38^. Although the relationship between isomiR expression and disease progression is not yet clearly understood, it has been reported that isomiRs might act as regulatory molecules and are associated with target mRNA repression^3^. In this study, we provide a systematic view of the molecular signatures of liver cancer-specific isomiRs and identify isomiR-21-5p | ±1 as a potent pro-tumorigenic isomiR in the development of liver cancer. This observation, for the first time, demonstrates an underlying mechanism by which hnRNPC causes isomiR-21-5p | ±1, which can inactivate GHR, contributing to the malignant transformation and growth of tumor cells in hepatocarcinogenesis.

miRNA sequencing studies have shown that 5′- and 3′-isomiRs are widespread and represent approximately 50% of miRNA copies in cells and tissues^39^. Because the 5′-end of a miRNA determines its seed sequence, 5′-isomiRs have an altered set of targets, rewiring functional networks in cells^40^. We identified eight isomiRs that were significantly dysregulated in liver cancer; however, only two isomiR-21-5p | ±1 were significantly associated with patients′ survival (Fig. 2b, e). These clinical associations were validated with a series of molecular and functional assays, indicating that isomiRs are not just byproducts of miRNA processing but also functional miRNAs that regulate their targets in the cancer development process. Although isomiRs of pri-miR-21 have been previously detected in other cancers^41,42^, they appear to be 3′-isomiRs, all with the same seed sequence, rather than 5′-isomiRs.

Because isomiRs differ by only a few nucleotides, examination of individual isomiR abundance and function requires careful attention to the specificity of our experimental techniques. First, RT–qPCR needs sufficient specificity to distinguish between the isomiRs for accurate determination of their abundance. Llorens et al. verified the specificity of 5′-isomiR detection using RT–qPCR with two specific primers^43^ and we found that the use of only one specific primer could distinguish between isomiRs with different 5′-ends (Fig. 3 and Supplementary Fig. 3). Next, antagomir specificity must be guaranteed for the isomiR functional assay. Many modification methods have been developed and applied to increase antagomir efficiency, but these modifications have lowered specificity^44^. Nevertheless, because *GHR* has target sites for both isomiR-21-5p | ±1 and miR-21-5p (Fig. 7e and Supplementary Fig. 7c), the results of our antagomir-based functional assays were mainly derived from the functional effects of isomiR-21-5p | ±1.

Although a handful of isomiRs were dysregulated in our LIHC samples, there should be more oncogenic or tumor-suppressing isomiR candidates in other cancers in which RBPs, such as hnRNPC and U2AF2, are dysregulated. In fact, hnRNPC and U2AF2 had strong eCLIP signals on 4 and 24 pri-miRNAs, respectively, in HepG2 cells (Supplementary Fig. 6b), suggesting that the cross-talk between miRNA processing and RBPs could be more prevalent than we anticipated. Some RBPs involved in miRNA maturation have been previously reported. For example, the DEAD-box RNA helicase subunit DDX17 promotes Drosha-mediated pri-miRNA processing^45,46^, and SMADs, activated by TGFβ/BMP, recruit the Drosha complex by binding to a conserved sequence in the stem region of approximately 20 miRNAs^47,48^. HIF interacts with Drosha to regulate pri-miR-215 processing under hypoxic conditions^49^. However, these studies only focused on the RBP-mediated regulation of Drosha processing efficiency and not specificity. Control of isomiR production by other RBPs requires further examination.

However, although we failed to detect any somatic mutations or RNA editing in pri-miRNAs associated with the production of isomiRs in liver cancer, such features could be present in other cancers. Alterations in cis-acting elements related to Drosha processing specificity can modulate the production ratio of oncogenic and tumor-suppressing isomiRs. In addition, the epitranscriptomic regulator METTL3/14 was previously reported to alter pri-miRNA structures by generating N6-methyladenosine (m6A) marks on RNAs, some of which are likely to affect Drosha processing independently of m6A reader proteins^50,51^. However, mutations in intrinsic factors are restricted to a few miRNAs in each individual, and somatic mutations, RNA editing, and RNA modifications affecting miRNA processing are sporadically detected. To comprehensively summarize the mutations in cis-acting elements, more samples should be investigated.

hnRNPC and U2AF2 compete for common binding sites, specifically single-stranded structures within polypyrimidine tracts^51,52^, and regulate pre-mRNA splicing by associating with the spliceosome^53^. In addition, Agranat-Tamir et al. reported that spliceosomes can interact with microprocessors^54^. These studies support the possibility of direct competition between hnRNPC and U2AF2 to regulate isomiR-21-5p | ±1 biogenesis. In fact, our analysis of hnRNPC and U2AF2 eCLIP-seq data showed their colocalization in the pri-miR-21 flanking regions (Fig. 5a). The greater effect of hnRNPC on isomiR-21-5p | ±1 biogenesis that we observed (Fig. 5 and Supplementary Fig. 5) could be due to the dominant distribution of poly-(U), providing a stronger affinity for hnRNPC in the upstream flanking region of pre-miR-21^52^. hnRNPC binds to pri-miR-21 over the basal junction and UG motif, which are thought to be anchored by Drosha^16^, and its effects on the regulation of the isomiR ratio could occur at any binding site in the 250-nt flanking region of pre-miR-21 because hnRNPC binds to an ~230-nt stretch of RNA^30–32^. However, the molecular mechanism by which hnRNPC regulates miRNA processing needs to be further clarified. There are two possible models: 1) hnRNPC binding to pri-miR-21 could interfere with Drosha binding, or 2) hnRNPC binding to pri-miR-21 could change the pri-miR-21 structure. Additional studies are required to evaluate these possibilities.

We report that isomiR-21-5p | ±1 regulates *GHR*, possibly acting as a tumor suppressor that is often downregulated in cancers. In fact, the deletion of Akt isoforms was previously observed to reduce the level of Ghr mRNA, mediating HCC progression via the insulin pathway in mice^55^, which suggests a possible mechanism for the tumor-suppressive function of GHR. The anti-growth effect of treatment with either AS-isomiR-21-5p | +1 or AS-isomiR-21-5p | -1 in all tested cells was phenocopied by treatment with a GHR-expressing plasmid (Supplementary Fig. 8a). Notably, *GHR* expression was suppressed in most cancers, with a few exceptions in the TCGA dataset (Supplementary Fig. 8b). Among them, *GHR* expression in LIHC and kidney renal clear cell carcinoma (KIRC) is clinically relevant, with high *GHR* expression associated with significant positive effects on overall survival but with negative effects in bladder urothelial carcinoma (BLCA), thyroid cancer (THCA), and stomach adenocarcinoma (STAD) (Supplementary Fig. 8c). In particular, *GHR* was most repressed in liver cancer compared to its expression in non-tumor tissues. The change in *GHR* expression was further validated with the Catholic_LIHC and Tsinghua_LIHC datasets (Supplementary Fig. 8d). Liver cancer patients with high *GHR* expression showed better overall prognosis and longer relapse-free survival (Supplementary Fig. 8e). However, the survival rate of patients with low expression of isomiR-21-5p or high expression of GHR dramatically decayed after four years, indicating that the isomiR-21-5p–GHR regulatory axis is no longer sufficient to maintain the benefits to patients in later years. Despite these results, further research is needed to examine whether GHR functions as a tumor suppressor and whether GHR depletion leads to tumor development in vivo.

In conclusion, our study not only sheds light on the biological and clinical importance of isomiRs but also has clinical implications for controlling miRNA and exogenous siRNA processing to produce intended miRNAs and siRNAs in the future.

## Supplementary information

Supplementary Information

## Data Availability

miRNA sequencing data and RNA-seq data were deposited in the NCBI Gene Expression Omnibus (http://www.ncbi.nlm.nih.gov/geo/) under the accession codes GSE174608 and GSE114564. All Python and R codes used in this study are available at the GitHub repository (https://github.com/ParkSJ-91/isomiR).

## References

[1] Bartel DP (2018). Metazoan microRNAs. Cell.

[2] Chiang HR (2010). Mammalian microRNAs: experimental evaluation of novel and previously annotated genes. Genes Dev..

[3] Cloonan N (2011). MicroRNAs and their isomiRs function cooperatively to target common biological pathways. Genome Biol..

[4] Tan GC (2014). 5’ isomiR variation is of functional and evolutionary importance. Nucleic Acids Res..

[5] Salem O (2016). The highly expressed 5’isomiR of hsa-miR-140-3p contributes to the tumor-suppressive effects of miR-140 by reducing breast cancer proliferation and migration. BMC Genomics.

[6] Han J (2004). The Drosha-DGCR8 complex in primary microRNA processing. Genes Dev..

[7] Denli AM, Tops BB, Plasterk RH, Ketting RF, Hannon GJ (2004). Processing of primary microRNAs by the Microprocessor complex. Nature.

[8] Han J (2006). Molecular basis for the recognition of primary microRNAs by the Drosha-DGCR8 complex. Cell.

[9] Fang W, Bartel DP (2015). The menu of features that define primary microRNAs and enable de novo design of microRNA genes. Mol. Cell.

[10] Roden C (2017). Novel determinants of mammalian primary microRNA processing revealed by systematic evaluation of hairpin-containing transcripts and human genetic variation. Genome Res..

[11] Zeng Y, Yi R, Cullen BR (2005). Recognition and cleavage of primary microRNA precursors by the nuclear processing enzyme Drosha. EMBO J..

[12] Ma H, Wu Y, Choi JG, Wu H (2013). Lower and upper stem-single-stranded RNA junctions together determine the Drosha cleavage site. Proc. Natl Acad. Sci. USA.

[13] Auyeung VC, Ulitsky I, McGeary SE, Bartel DP (2013). Beyond secondary structure: primary-sequence determinants license pri-miRNA hairpins for processing. Cell.

[14] Nguyen TA (2015). Functional anatomy of the human microprocessor. Cell.

[15] Kim K, Nguyen TD, Li S, Nguyen TA (2018). SRSF3 recruits DROSHA to the basal junction of primary microRNAs. RNA.

[16] Kwon SC (2019). Molecular basis for the single-nucleotide precision of primary microRNA processing. Mol. Cell.

[17] Bofill-De Ros X (2019). Structural differences between pri-miRNA paralogs promote alternative drosha cleavage and expand target repertoires. Cell Rep..

[18] Macrae IJ (2006). Structural basis for double-stranded RNA processing by Dicer. Science.

[19] MacRae IJ, Zhou K, Doudna JA (2007). Structural determinants of RNA recognition and cleavage by Dicer. Nat. Struct. Mol. Biol..

[20] Park JE (2011). Dicer recognizes the 5’ end of RNA for efficient and accurate processing. Nature.

[21] Gu S (2012). The loop position of shRNAs and pre-miRNAs is critical for the accuracy of dicer processing in vivo. Cell.

[22] Kim H (2019). Bias-minimized quantification of microRNA reveals widespread alternative processing and 3’ end modification. Nucleic Acids Res..

[23] Telonis AG (2017). Knowledge about the presence or absence of miRNA isoforms (isomiRs) can successfully discriminate amongst 32 TCGA cancer types. Nucleic Acids Res..

[24] Marrero JA (2018). Diagnosis, staging, and management of hepatocellular carcinoma: 2018 practice guidance by the american association for the study of liver diseases. Hepatology.

[25] Nam JW (2014). Global analyses of the effect of different cellular contexts on microRNA targeting. Mol. Cell.

[26] Yang Y (2017). Recurrently deregulated lncRNAs in hepatocellular carcinoma. Nat. Commun..

[27] Friedlander MR, Mackowiak SD, Li N, Chen W, Rajewsky N (2012). miRDeep2 accurately identifies known and hundreds of novel microRNA genes in seven animal clades. Nucleic Acids Res..

[28] Wang AG (2005). Gender-dependent hepatic alterations in H-ras12V transgenic mice. J. Hepatol..

[29] Consortium EP (2012). An integrated encyclopedia of DNA elements in the human genome. Nature.

[30] Huang M (1994). The C-protein tetramer binds 230 to 240 nucleotides of pre-mRNA and nucleates the assembly of 40S heterogeneous nuclear ribonucleoprotein particles. Mol. Cell Biol..

[31] McAfee JG, Soltaninassab SR, Lindsay ME, LeStourgeon WM (1996). Proteins C1 and C2 of heterogeneous nuclear ribonucleoprotein complexes bind RNA in a highly cooperative fashion: support for their contiguous deposition on pre-mRNA during transcription. Biochemistry.

[32] McCloskey A, Taniguchi I, Shinmyozu K, Ohno M (2012). hnRNP C tetramer measures RNA length to classify RNA polymerase II transcripts for export. Science.

[33] Agarwal V, Bell GW, Nam JW, Bartel DP (2015). Predicting effective microRNA target sites in mammalian mRNAs. Elife.

[34] Lewis BP, Burge CB, Bartel DP (2005). Conserved seed pairing, often flanked by adenosines, indicates that thousands of human genes are microRNA targets. Cell.

[35] Krek A (2005). Combinatorial microRNA target predictions. Nat. Genet..

[36] Brennecke J, Stark A, Russell RB, Cohen SM (2005). Principles of microRNA-target recognition. PLoS Biol..

[37] Kim D (2016). General rules for functional microRNA targeting. Nat. Genet..

[38] Neilsen CT, Goodall GJ, Bracken CP (2012). IsomiRs-the overlooked repertoire in the dynamic microRNAome. Trends Genet..

[39] McCall MN (2017). Toward the human cellular microRNAome. Genome Res..

[40] Manzano M, Forte E, Raja AN, Schipma MJ, Gottwein E (2015). Divergent target recognition by coexpressed 5’-isomiRs of miR-142-3p and selective viral mimicry. RNA.

[41] Telonis AG, Rigoutsos I (2018). Race disparities in the contribution of miRNA isoforms and tRNA-derived fragments to triple-negative breast cancer. Cancer Res..

[42] Koppers-Lalic D (2016). Noninvasive prostate cancer detection by measuring miRNA variants (isomiRs) in urine extracellular vesicles. Oncotarget.

[43] Llorens F (2013). A highly expressed miR-101 isomiR is a functional silencing small RNA. BMC Genomics.

[44] Lennox KA, Behlke MA (2011). Chemical modification and design of anti-miRNA oligonucleotides. Gene Ther..

[45] Mori M (2014). Hippo signaling regulates microprocessor and links cell-density-dependent miRNA biogenesis to cancer. Cell.

[46] Moy RH (2014). Stem-loop recognition by DDX17 facilitates miRNA processing and antiviral defense. Cell.

[47] Davis BN, Hilyard AC, Lagna G, Hata A (2008). SMAD proteins control DROSHA-mediated microRNA maturation. Nature.

[48] Davis BN, Hilyard AC, Nguyen PH, Lagna G, Hata A (2010). Smad proteins bind a conserved RNA sequence to promote microRNA maturation by Drosha. Mol. Cell.

[49] Hu J (2016). MiR-215 is induced post-transcriptionally via HIF-drosha complex and mediates glioma-initiating cell adaptation to hypoxia by targeting KDM1B. Cancer Cell.

[50] Alarcon CR, Lee H, Goodarzi H, Halberg N, Tavazoie SF (2015). N6-methyladenosine marks primary microRNAs for processing. Nature.

[51] Liu N (2015). N(6)-methyladenosine-dependent RNA structural switches regulate RNA-protein interactions. Nature.

[52] Zarnack K (2013). Direct competition between hnRNP C and U2AF65 protects the transcriptome from the exonization of Alu elements. Cell.

[53] Wahl MC, Will CL, Luhrmann R (2009). The spliceosome: design principles of a dynamic RNP machine. Cell.

[54] Agranat-Tamir L, Shomron N, Sperling J, Sperling R (2014). Interplay between pre-mRNA splicing and microRNA biogenesis within the supraspliceosome. Nucleic Acids Res..

[55] Wang Q (2016). Spontaneous hepatocellular carcinoma after the combined deletion of Akt isoforms. Cancer Cell.

