# Supplementary material for: hnRNPC induces isoform shifts in miR-21-5p leading to cancer development
Source: Exp Mol Med. 2022 Jun 21;54(6):812–24. doi: 10.1038/s12276-022-00792-2 (PMC9256715; doi:10.1038/s12276-022-00792-2)
Supplement: Supplementary file 1 — Supplementary Information [file 12276_2022_792_MOESM1_ESM.pdf]

# - Supplementary Information -

hnRNPC induces isoform shifts in miR-21-5p leading to cancer development

Seokju Park[1,9], Hee Doo Yang[2,4,9], Jwa-Won Seo[8], Jin-Wu Nam[1,3,7*] and Suk Woo Nam[2,4,5,6,*]

[1]Department of Life Science, College of Natural Sciences, Hanyang University, Wangsimni-ro 222, Seongdong-gu, Seoul 04763, Republic of Korea
[2] Department of Pathology, College of Medicine, The Catholic University of Korea, Banpo-daero 222, Seocho-gu, Seoul 06591, Republic of Korea
[3]Research Institute for Convergence of Basic Sciences, Hanyang University, Wangsimni-ro 222, Seongdong-gu, Seoul 04763, Republic of Korea
[4]Functional RNomics Research Center, The Catholic University of Korea, Banpo-daero 222, Seocho-gu, Seoul 06591, Republic of Korea
[5]Department of Biomedicine & Health Sciences, Graduate School, The Catholic University of Korea, Seoul, 06591, Korea.
[6]NEORNAT Inc., The Catholic University of Korea, 222 Banpo-daero, Seocho-gu, Seoul, Republic of Korea
[7]Bio-BigData Center, Hanyang Institute for Bioscience and Biotechnology, Hanyang University, Wangsimni-ro 222, Seongdong-gu, Seoul 04763, Republic of Korea
[8]Department of Information Systems, University of Maryland, Baltimore County, MD 20742, USA

[9]These authors contributed equally to this paper.
[*]Corresponding authors: jwnam@hanyang.ac.kr, swnam@catholic.ac.kr

Supplementary Materials and Methods

Processing of high-throughput sequencing data. All RNA-seq data were preprocessed to remove adaptor sequences from reads using seqtk (version 1.0) and to trim parts of reads at positions where the quality fell below a minimum of 20 using Sickle (version 1.2, parameters: -q 20 -l 20). Reads less than 20 nt in length were removed. Preprocessed RNA-seq reads were mapped to the reference genome using Bowtie (version 1.2.2) with default parameters. The mismatch rates at each position of the reads were measured so that the shorter section of the read adjacent to positions where the mismatch rate was more than 10% were further trimmed. The resulting reads were mapped to transcriptomes (GENCODE v19) and the gene expression levels were calculated with reads per kilobase of transcript per million mapped reads (RPKM) using the Bitseq VB algorithm. To measure the gene expression level, the expression values of all isoforms were summed and normalized by quantile normalization. On the other hand, adaptor sequences were trimmed from miRNA-seq reads using cutadapt (version 1.18, parameter: --overlap=6 -a TGGAATTCTCGGGTGCCAAGG -m 18 -M 26) and the 3′-ends of reads with a minimum quality ≤ 20 were removed using Sickle (version 1.2, parameters: -q 20 -l 18 -x). The levels of miRNAs and isomiRs (with 5′-ends positioned from 5 nt upstream to 5 nt downstream of the 5′-end of the corresponding miRNA, annotated in miRBase) were quantified using the miRDeep2 algorithm and an in-house script. Briefly, all preprocessed reads were collapsed using a mapper module (parameters: -e -h -j -l 18 -m -s) and then mapped to miRNA precursor sequences with flanking regions, downloaded from miRBase (version 21) using a quantifier module (parameters: -d -g 2). Multi-mapped reads were normalized by the total mapped reads for each precursor miRNA. The expression levels (RPM) of all miRNAs and isomiRs were calculated by only considering perfectly matched reads. The expression level was calculated by collapsing all of the different 3′-ends and normalized using quantile normalization. miRNAs/isomiRs and genes with greater than 1 RPM or 1 RPKM, respectively, were kept and considered to be expressed. The isomiR ratio for miRNAs was calculated as the ratio of reads assigned to isomiRs over reads assigned to their corresponding canonical miRNAs.

Re-annotation of Drosha and Dicer cleavage sites. To allow careful checking of the 5′-ends of miRNAs in miRBase, Drosha- and Dicer-independent miRNAs were first excluded by analyzing public miRNA-seq data from Drosha- or Dicer-deficient cells [1]. Only miRNAs for which there was a greater than four-fold decrease in reads in the Drosha- and Dicer-deficient conditions were considered. If the 5′-end of more than 50% of the reads for a locus differed from that in the miRBase (version 21) in more than 90% of all samples and in more than 2 cohorts, the reads with that 5′-end were regarded to be canonical miRNAs in the liver and the others were considered to be 5′-isomiRs. Otherwise, we followed the miRBase annotations.

Prioritization of isomiR-target interactions. Our overall pipeline is displayed in Fig. 2B. Briefly, to predict targets of miRNAs and isomiRs (interactions between miRNAs and targets), the current state-of-the-art program, TargetScan v7.0, was run with the predicted 3′-UTR landscape in Huh7 cells. If there were multiple isoforms to a gene, a representative isoform that was most abundant was selected. Next, to find tumorigenesis-associated genes and miRNAs/isomiRs, we conducted one-way ANOVA tests on the isomiR ratios and Wilcoxon signed rank-sum tests on miRNA/isomiR and gene expression level differences between non-tumor and HCC samples. Among the DEGs, only genes related to cancer pathways were left behind using GO terms. To associate gene expression with clinical outcomes, uni- and multi-variate survival analyses on miRNA/isomiR and miRNA families with the same seed sequence, and mRNAs were analyzed with all clinical variables including overall survival and relapse-free survival in the Catholic_LIHC and TCGA_LIHC cohorts (5-year survival). By intersecting the results from all of the above analyses, the top prioritized miRNA/isomiR-target interactions between mRNAs and miRNAs/isomiRs were obtained.

Cis-motif analysis related to isomiR ratios. The mean isomiR ratio (only ±1 offsets were considered for convenience of analysis) was calculated using miRNAs profiled in Catholic_LIHC and Tsinghua_LIHC but not TCGA_LIHC, because isomiR ratios from TCGA_LIHC displayed lower correlations with those of the other cohorts. To infer RNA structure, we used two steps to predict the secondary structure of the pre-miRNA sequence with 20-nt flanking region. The secondary structure of the pre-miRNA sequence was predicted using RNAsubopt (parameters: -e 5 -s -d2 –noLP) in the Vienna RNA package [2]. We chose the structure with the lowest minimum free energy with only one loop. Then, the pre-miRNA sequence with 20-nt flanking region was folded in a constrained way though the pre-miRNA structure using RNAsubopt (with the same parameters). Using the folded structures, the mismatch proportion at each position and known motifs were compared between the two groups, divided by the mean isomiR ratio. The statistical significance was tested using Fisher′s exact test.

Mutation and RNA editing analysis
To test whether isomiR expression changes result from alterations in the pri-miRNA sequence or structural alterations caused by genomic variations or RNA editing, all somatic mutations and RNA editing events in pri-miR-21 were examined from whole genome sequencing, RNA-seq, and miRNA-seq data from TCGA using the GATK pipeline [3,4], DREAM [5] and GIREMI [6] packages.

Analysis of eCLIP-seq data. All "bed narrowPeak" files processed from eCLIP-seq data (submitted by April 29, 2018) were downloaded. Among the peaks with the following criteria, $\log_2$ fold-change of ≥ 1 and FDR ≤ 0.05, RBPs with the peaks within the 100-nt flanking region of pre-mir-21 were used for this study.

MTT assay. Cells were seeded in a 12-well plate for transfection. After transfection, cells were incubated with 0.5 mg/ml of MTT [3-(4,5-dimethylthiazol-2-yl)-2,5-diphenyltetrazolium bromide] solution (Sigma) for 1 hr. The dark blue formazan products formed by viable cells were dissolved in dimethyl sulfoxide (DMSO; Sigma), and the absorbance was measured using a VICTOR3 Multilabel plate reader (PerkinElmer).

Western blots. Cells were lysed using a lysis buffer (50 mM HEPES, 5 mM EDTA, 50 mM NaCl, 1% Triton X-100, 50 mM NaF, 10 mM $Na_2P_4O_7$, 1 mM Na_3VO_4, 5 ug/mL aprotinin, 5 ug/mL leupeptin, 1 mM PMSF, and a protease inhibitor cocktail). Lysates containing equal amounts of proteins were separated by SDS-PAGE and transferred onto a polyvinylidene difluoride membrane (Bio-Rad). The blots were blocked with a 5% skim milk solution and incubated with the following antibodies: anti-hnRNPC, anti-GHR, and anti-GAPDH (Santa Cruz Biotechnology). The Immobilon™ Western blot detection system (Millipore) was used to detect bound antibodies. The intensities of the Western blot bands were quantified using LAS-4000 (Fuji Photo Film Co.).

Plasmid source. The GHR (OHu20603) and hnRNPC (OHu04412) expression plasmids were purchased from Genscript.

Luciferase reporter plasmids and assays. The 3′-UTR of the *GHR* mRNA was PCR amplified from cDNAs from the HCC cell lines and cloned into the Xho I/Not I sites of the psiCHECK-2 vector (Promega), downstream of the Renilla luciferase gene, according to the manufacturer′s instructions. Luciferase activity was assayed using the Dual-Luciferase Reporter Assay System (Promega) according to the manufacturer′s instructions. The expression of Renilla luciferase was normalized with that of co-expressed firefly luciferase.

FLAG immunoprecipitation. HCC cells were transfected with pCMV-MIR_mir-21 and pcDNA3.1_hnRNPC, which encodes a FLAG-tagged version of hnRNPC. 48 h after transfection, the cells were washed with phosphate-buffered saline (PBS) and lysed at 4°C in PBS, pH 7.2, containing 1.0% NP-40, 0.5% sodium deoxycholate, 0.1% SDS, 10mM NaF, 1.0 mM NaVO4, and 1.0% protease inhibitor cocktail (Sigma). The FLAG tag was immunoprecipitated with anti-FLAG DynaBeads (Invitrogen) during an overnight incubation. Immunoprecipitated proteins were eluted using 3X FLAG peptide (Sigma) and analyzed by Western blot, probing with anti-FLAG antibody (Cell Signaling). For pre-mir-21 expression analysis using qRT-PCR, RNA was isolated and reverse-transcribed using a miScript II RT kit (Qiagen).

HCC mouse model. H-*ras*12V transgenic mice (*Ras*-Tg mice) were kindly provided by Dr. Dae-Yeoul Yu (Laboratory of Human Genomics, Korea Research Institute of Bioscience and Biotechnology) [7]. Male mice spontaneously developed HCC beginning at approximately 10~15 weeks of age. Livers were then harvested from the mice at 25 weeks of age and processed for analysis. All animal experiments were undertaken in accordance with the National Institutes of Health′s Guide for the Care and Use of Laboratory Animals, with approval of the Animal Experiment Ethics Committee of the Catholic University of Korea College of Medicine.

Supplementary Figures

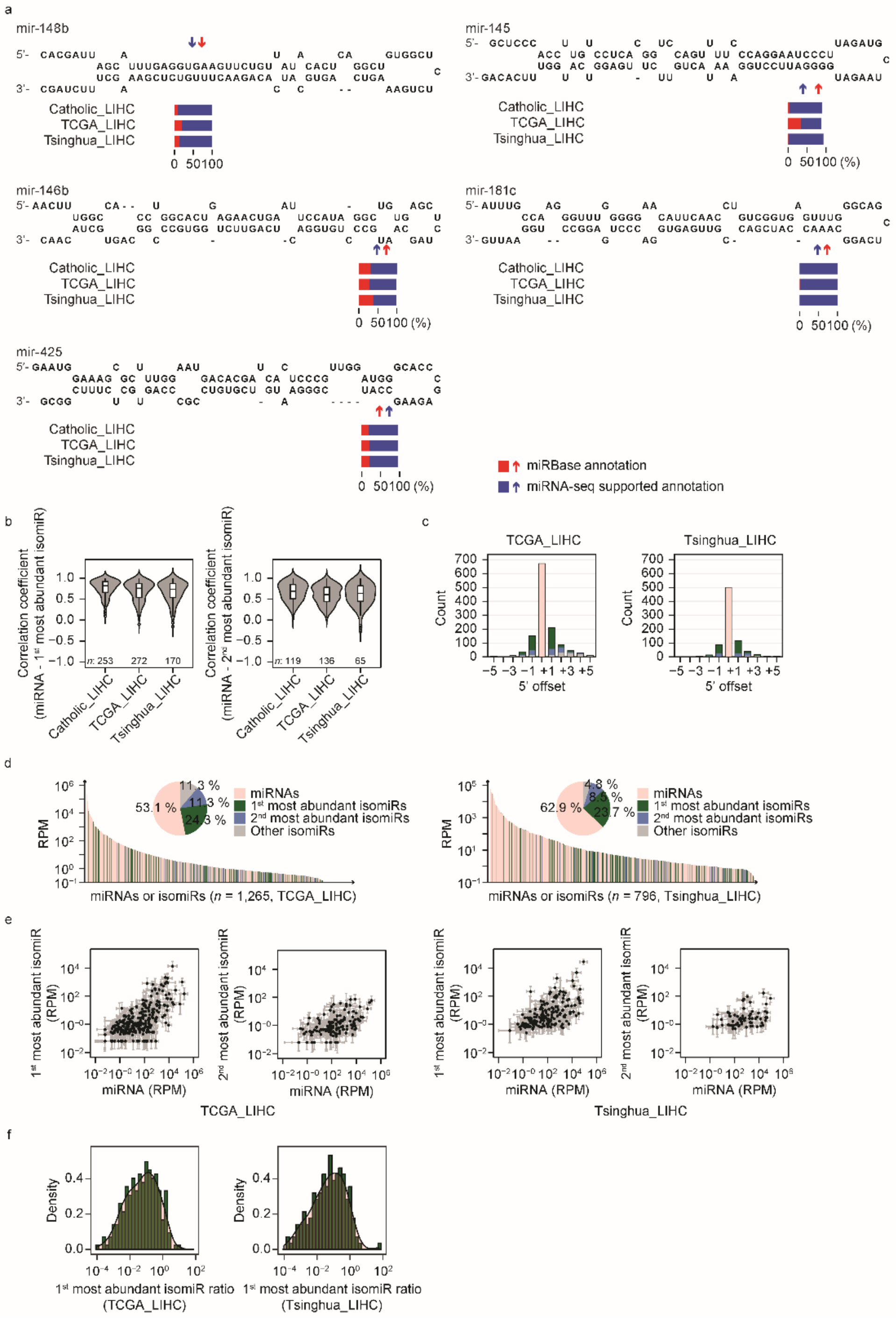

Supplementary Fig. 1 Expression patterns of isomiRs and their relationship with the associated miRNAs. a Examples of miRNAs with re-annotated 5′-ends. Red arrows indicate miRBase-annotated cleavage sites and blue arrows indicate miRNA-seq-supported cleavage sites. The median proportions of the miRNA-seq-supported reads and miRbase annotated miRNA reads are drawn in the same color scheme.

b Distribution of correlation coefficients between the isomiR and miRNA expression levels in each cohort. Left plot, the most abundant isomiRs; right plot, the second most abundant isomiRs. **c** Numbers of expressed miRNAs and isomiRs in TCGA_LIHC and Tsinghua_LIHC. **d** The pool of expressed miRNAs/isomiRs ordered by their expression level in TCGA_LIHC (left) and Tsinghua_LIHC (right). The y-axis indicates the median RPM of a given miRNA/isomiR among all samples. The inset showed the relative abundance of miRNAs and isomiRs. **e** Comparison of the differences in expression levels between miRNAs and the most abundant isomiRs and between miRNAs and the second most abundant isomiRs in TCGA_LIHC (left) and Tsinghua LIHC (right). Data are represented as median ± SD. **f** Density plots of the mean ratio of the most abundant isomiRs in TCGA_LIHC (left) and Tsinghua_LIHC (right).

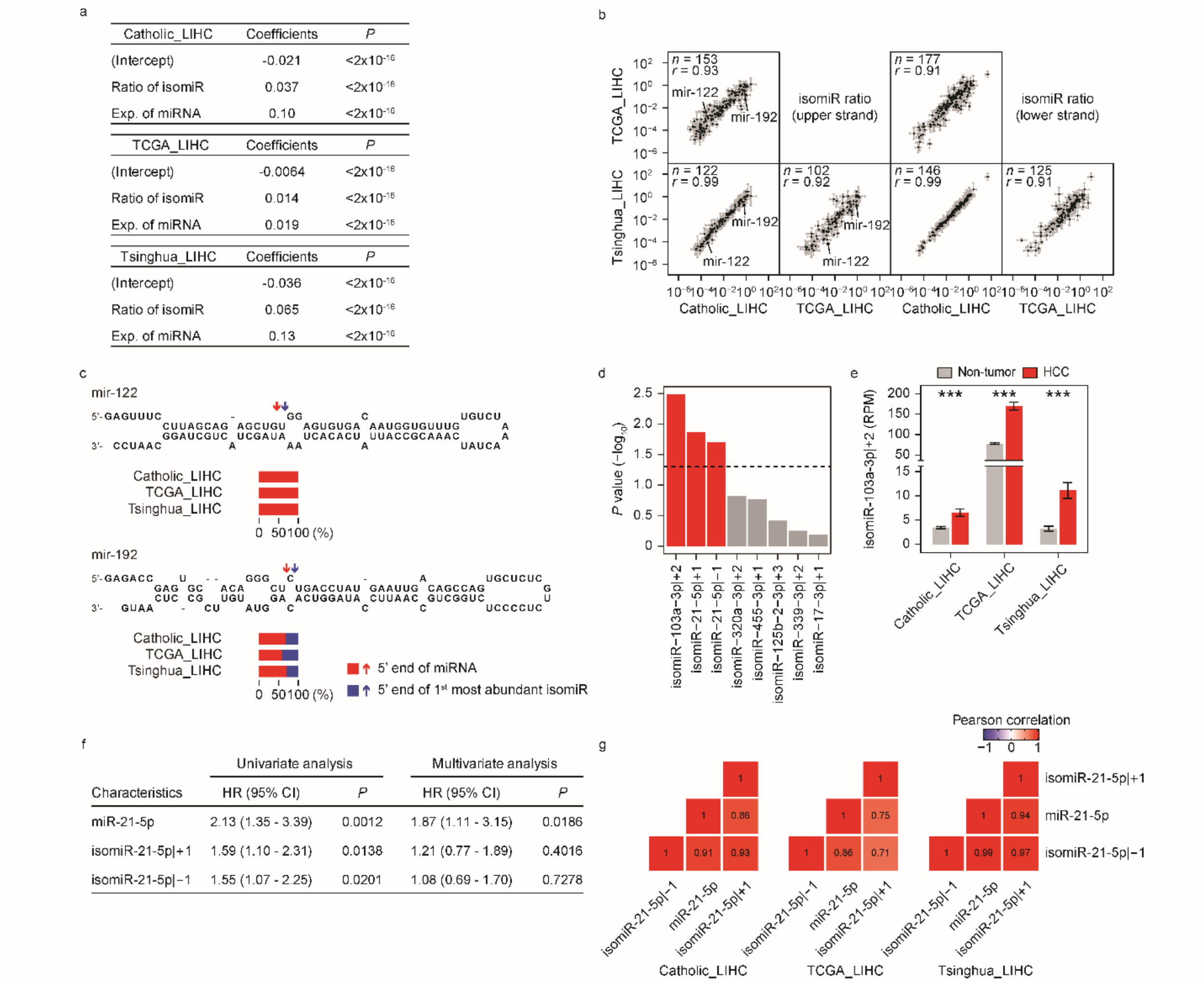

Supplementary Fig. 2 Extended identification and characterization of isomiR-21-5p|±1. a A multivariate linear regression model was built to account for the correlations between the isomiR expression level and other factors, including the expression level of the corresponding miRNAs and the isomiR ratios. **b** Comparison of isomiR ratios between cohorts. The right three plots show miRNAs/isomiRs that were generated from the upper strand of the pri-miRNA and the left three plots show those generated from lower strand. **c** Examples of isomiRs (miR-122-5p and miR-192-5p) associated with different isomiR ratios. Red arrows indicate the position of the 5′-ends of the miRNAs and blue arrows indicate the 5′-ends of the most abundant isomiRs. The insets represent the median proportions of miRNA-seq reads for the miRNAs and isomiRs and are drawn in the same color scheme. **d** Univariate analysis of the effects of the expression levels of isomiRs, which showed differential expression and isomiR ratios in liver cancer samples, on overall survival. **e** Comparison between the isomiR-103a-3p|+2 expression level in non-tumor and HCC samples in each cohort. **f** Uni- and multivariate analysis of the effects of the expression levels of miR-21-5p and its isomiRs on overall survival. **g** Correlation plots analyzing Pearson correlations between the mapped read counts of miR-21-5p and its isomiRs in each cohort. The colors indicate the strength of the correlation and the coefficients are indicated in boxes. Data represent the median ± SD in **b** and **e**. Statistical significance was determined by Wald test **d** and one-tailed Wilcoxon rank-sum test or one tailed Wilcoxon signed rank-sum test **e**, ***: FDR $\leq$ 0.001.

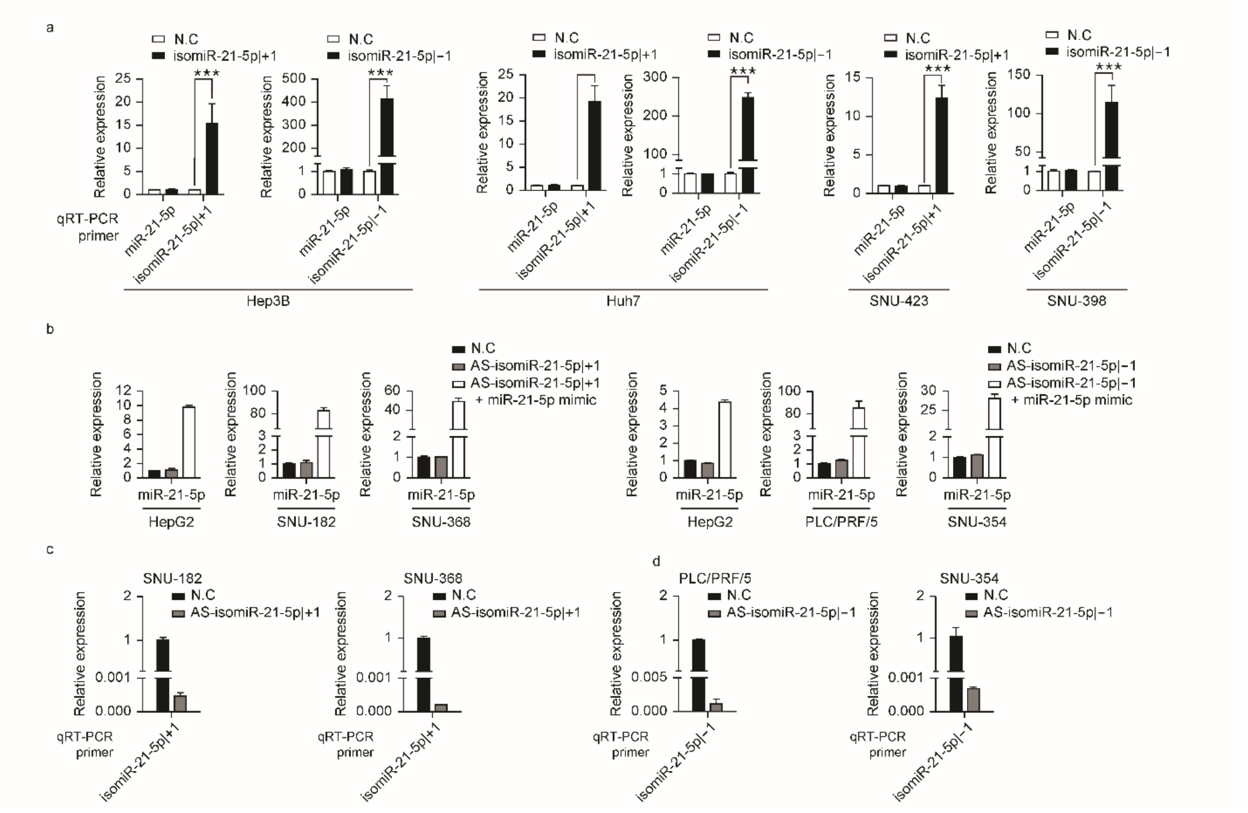

Supplementary Fig. 3 Extended validation of qRT-PCR and mimic assays for isomiR-21-5p|±1 quantification and perturbation. a Hep3B, Huh7, SNU-423, and SNU-398 cells were transfected with the isomiR-21-5p|+1 mimic or the isomiR-21-5p|-1 mimic, after which qRT-PCR was performed to quantify the levels of isomiR-21-5p|+1 and isomiR-21-5p|-1. **b** HepG2, PLC/PRF/5, SNU-354, SNU-182 and SNU-368 cells were transfected with miR-21-5p after transfecting the AS-isomiR-21-5p|+1 or the AS-isomiR-21-5p|-1, after which qRT-PCR was performed to quantify the levels of miR-21-5p. **c** SNU-182 and SNU-368 cells were transfected with AS-isomiR-21-5p|+1. Otherwise, as in Fig. 3c. **d** PLC/PRF/5 and SNU-354 cells were transfected with AS-isomiR-21-5p|-1. Otherwise, as in Fig. 3c. Data represent the median ± SEM of three independent experiments/three replicates. Statistical significance was determined by unpaired Students t-test, ***: $P \leq 0.001$.

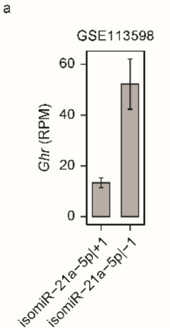

b

Group	No.	No. of tumor mass					
		17 wks	19 wks	21 wks	23 wks	24 wks	25 wks
WT	1	1	1	2	2	3	6
	2		2	3	4	6	7
	3		3	3	3	4	6
	4		1	2	2	5	10
	5						
AS-isomiR-21a-5p	1			2	2	3	5
	2					2	3
	3						
	4						

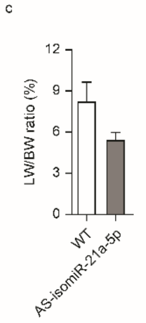

Supplementary Fig. 4 Extended findings related to targets of isomiR-21-5p|±1. a The expression levels of isomiR-21a-5p|+1 and isomiR-21a-5p|-1 in normal mouse liver. Data represent the median ± SD. **b** A table showing the number of tumor masses for each mouse at 17, 19, 21, 23, and 25 weeks of age. **c** Effect of treatment with AS-isomiR-21a-5p|±1 on the ratio of liver weight (LW) to body weight (BW) in the HCC mouse model. Data represent the mean ± SEM of three independent experiments/three replicates.

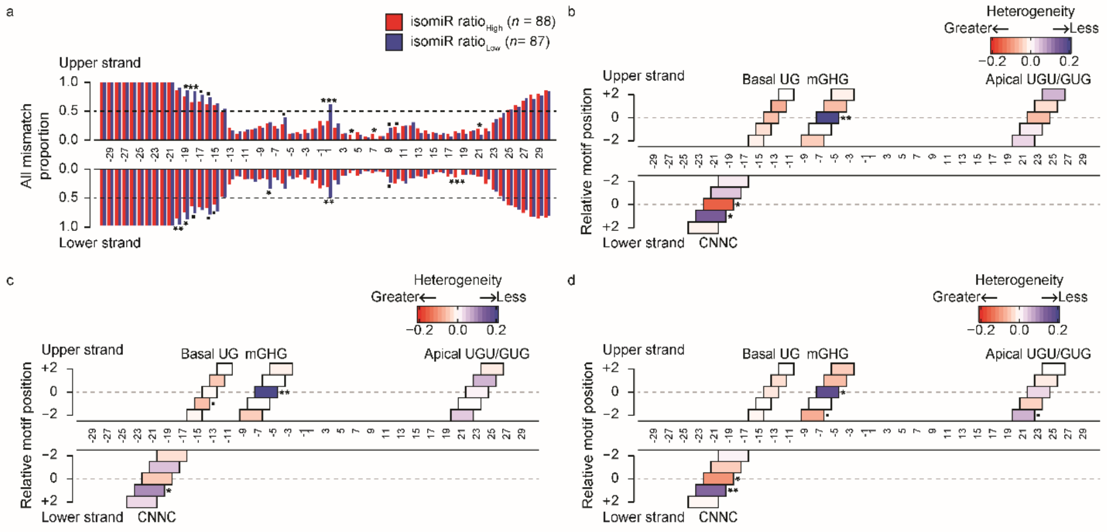

e

isomiR ratio ~	Exp. of mature miRNA		Exp. of isomiR		Exp. of *hnRNPC*		Exp. of host transcript	
	Coefficients	*P*	Coefficients	*P*	Coefficients	*P*	Coefficients	*P*
Catholic_LIHC	-0.36	0.0027	0.93	5.43×10^{-13}	0.39	7.75×10^{-6}	-0.16	0.072
TCGA_LIHC	-0.0079	0.76	0.69	3.97×10^{-31}	0.21	6.74×10^{-5}	-0.30	7.87×10^{-5}
Tsinghua_LIHC	-0.19	0.13	0.41	0.029	0.75	5.43×10^{-7}	-0.038	0.78

f

isomiR ratio ~	Exp. of mature miRNA		Exp. of isomiR		Exp. of *U2AF2*		Exp. of host transcript	
	Coefficients	*P*	Coefficients	*P*	Coefficients	*P*	Coefficients	*P*
Catholic_LIHC	-0.19	0.11	0.85	3.07×10^{-10}	0.30	0.0024	-0.24	0.013
TCGA_LIHC	-0.01	0.71	0.69	7.92×10^{-31}	0.23	1.09×10^{-4}	-0.28	2.04×10^{-4}
Tsinghua_LIHC	-0.076	0.60	0.31	0.18	0.48	2.53×10^{-4}	0.092	0.59

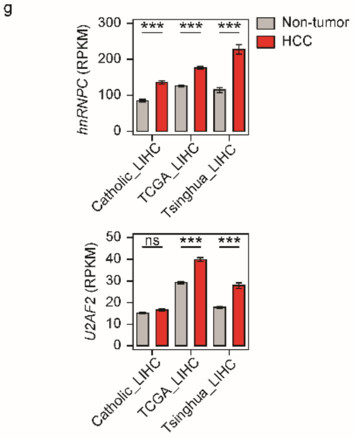

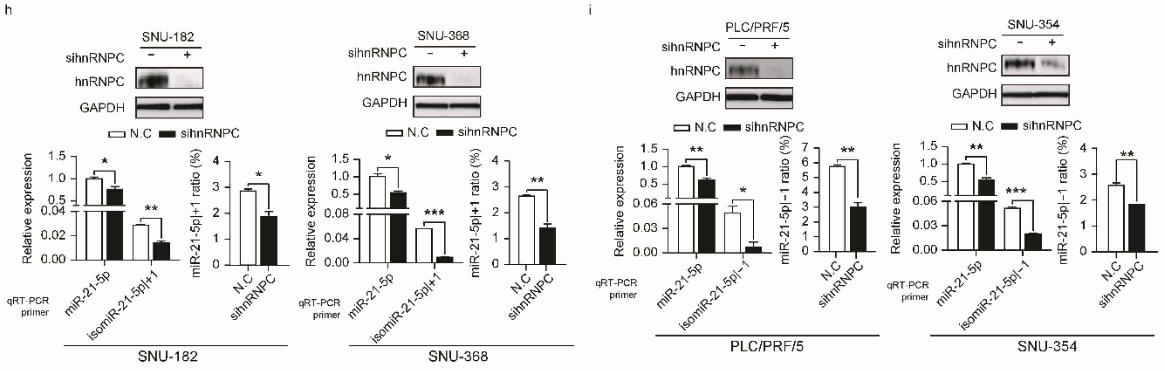

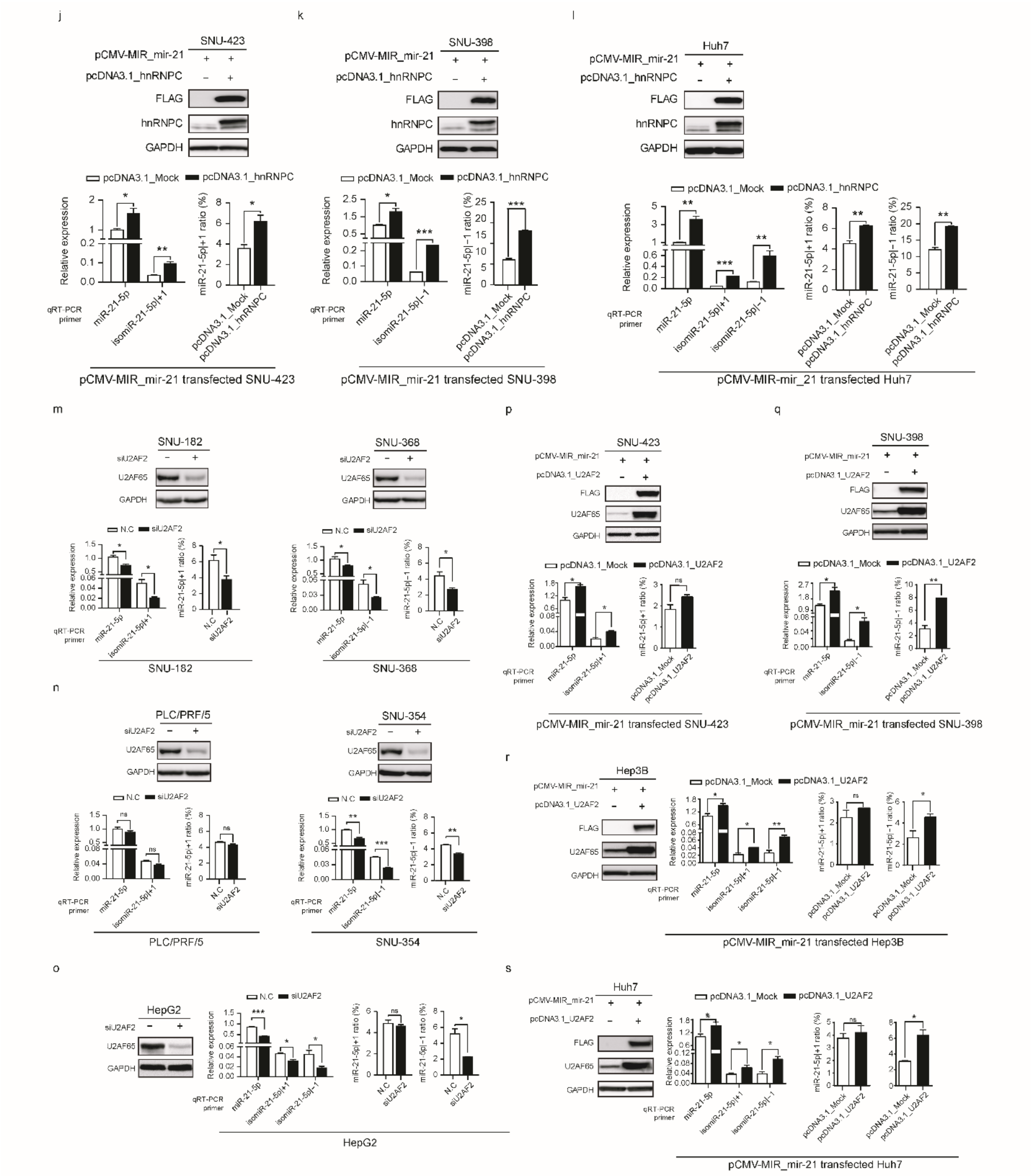

Supplementary Fig. 5 Extended analysis for the identification of cis- and trans-acting elements that regulate isomiR expression. a A comparison of mismatch proportions between low and high isomiR ratios at each position. **b**–**d** The log-odds ratio of the number of known motifs between miRNAs with low and high isomiR ratios (**b**), those with low and high isomiR|-1 ratios (**c**), and those with low and high isomiR|+1 ratios (**d**) are estimated as the impact on the 5′-heterogeneity of Drosha processing at each position. The known motifs are indicated in either upper strand or lower strand of miRNAs. The y-axis indicates the

distance from the ideal position for each motif. **e**, **f** Univariate and multivariate analysis of the expression levels of miR-21-5p, *VMP1*, *hnRNPC* (**e**), and *U2AF2* (**f**). **g** Comparisons between the expression levels of *hnRNPC* and *U2AF2* in non-tumor and HCC samples in each LIHC dataset (data represent the median ± SD; one-tailed Wilcoxon rank-sum test for Catholic and TCGA_LIHC and one-tailed Wilcoxon signed rank-sum test for Tsinghua_LIHC with paired data). **h**, **i** SNU-182 and SNU-368 (**h**) and PLC/PRF/5 and SNU-354 (**i**) were transfected with sihnRNPC. qRT-PCR was conducted to quantify the levels of miR-21-5p, isomiR-21-5p|+1, and isomiR-21-5p|-1, after which the isomiR ratios were calculated. **j**–**l** SNU-423 (**j**), SNU-398 (**k**), and Huh7 (**l**) cells were transfected with pcDNA3.1_hnRNPC in the pCMV-MIR-mir-21-transfected background. qRT-PCR was conducted to quantify the levels of miR-21-5p, isomiR-21-5p|+1, and isomiR-21-5p|-1, after which the isomiR ratios were calculated. **m**–**o** SNU-182 and SNU-368 (**m**), PLC/PRF/5 and SNU-354 (**n**), and HepG2 (**o**) cells were transfected with siU2AF2. qRT-PCR was conducted to quantify the levels of miR-21-5p, isomiR-21-5p|+1, and isomiR-21-5p|-1, after which the isomiR ratio was calculated. **p**–**s** SNU-423 (**p**), SNU-398 (**q**), Hep3B (**r**), and Huh7 (**s**) cells were transfected with pcDNA3.1_U2AF2 in a pCMV-MIR-mir-21-transfected background. qRT-PCR was conducted to quantify the levels of miR-21-5p, isomiR-21-5p|+1, and isomiR-21-5p|-1, after which the isomiR ratio was calculated. Data represent the mean ± SEM of three independent experiments/three replicates **h**–**s**. Statistical significance was determined by Fisher exact test **a**–**d**, unpaired Students t-test **h**–**s**, ■: FDR ≤ 0.1, *: FDR ≤ 0.05, **: FDR ≤ 0.01, and ***: FDR ≤ 0.001 for **a**–**d** and **g**, ns: no significance, *: P ≤ 0.05, **: P ≤ 0.01, and ***: P ≤ 0.001 for **h**–**s**.

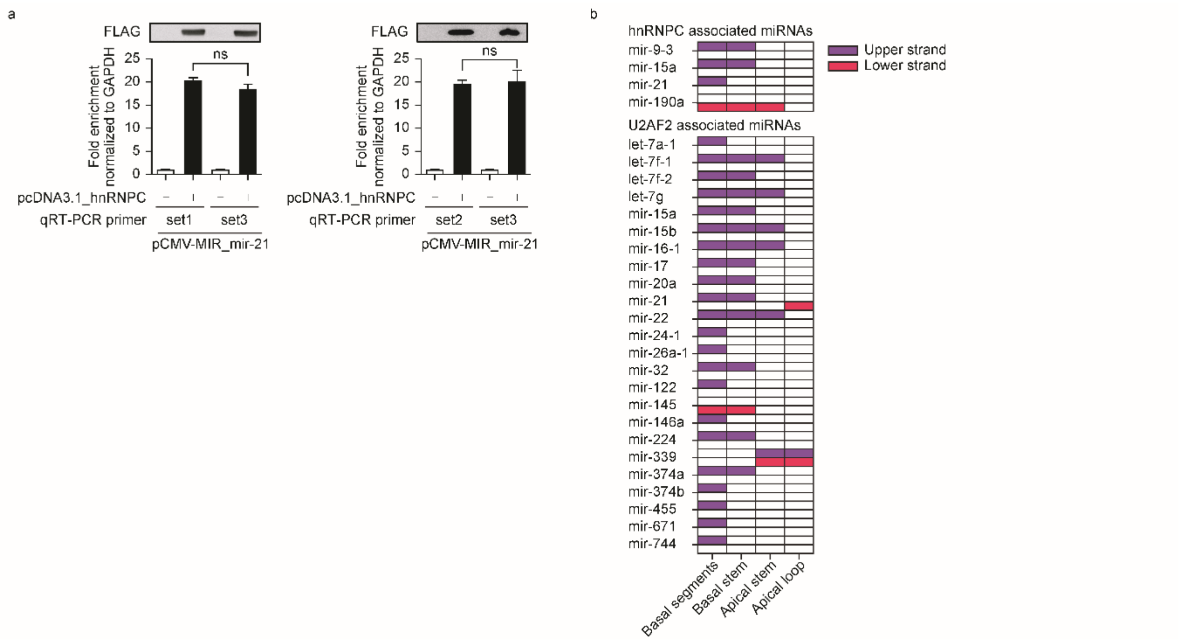

Supplementary Fig. 6 Substrates that can be controlled by hnRNPC and U2AF2. **a** Immunoprecipitation of FLAG-tagged hnRNPC was performed to investigate hnRNPC binding affinity to pri-mir-21. The levels of pri-mir-21 in the immunoprecipitated materials were determined by qRT-PCR using the three different sets of primers indicated in Fig. 6a. Hep3B cells were transfected with wt pCMV-MIR_mir-21 and pcDNA3.1_hnRNPC. Data represent the mean ± SEM of three independent experiments/three replicates. Statistical significance was determined by Student t-test, ns: no significance. **b** eCLIP-seq data supported binding sites of hnRNPC and U2AF2 in basal segment, basal stem, apical stem, and apical loop regions of pri-miRNAs.

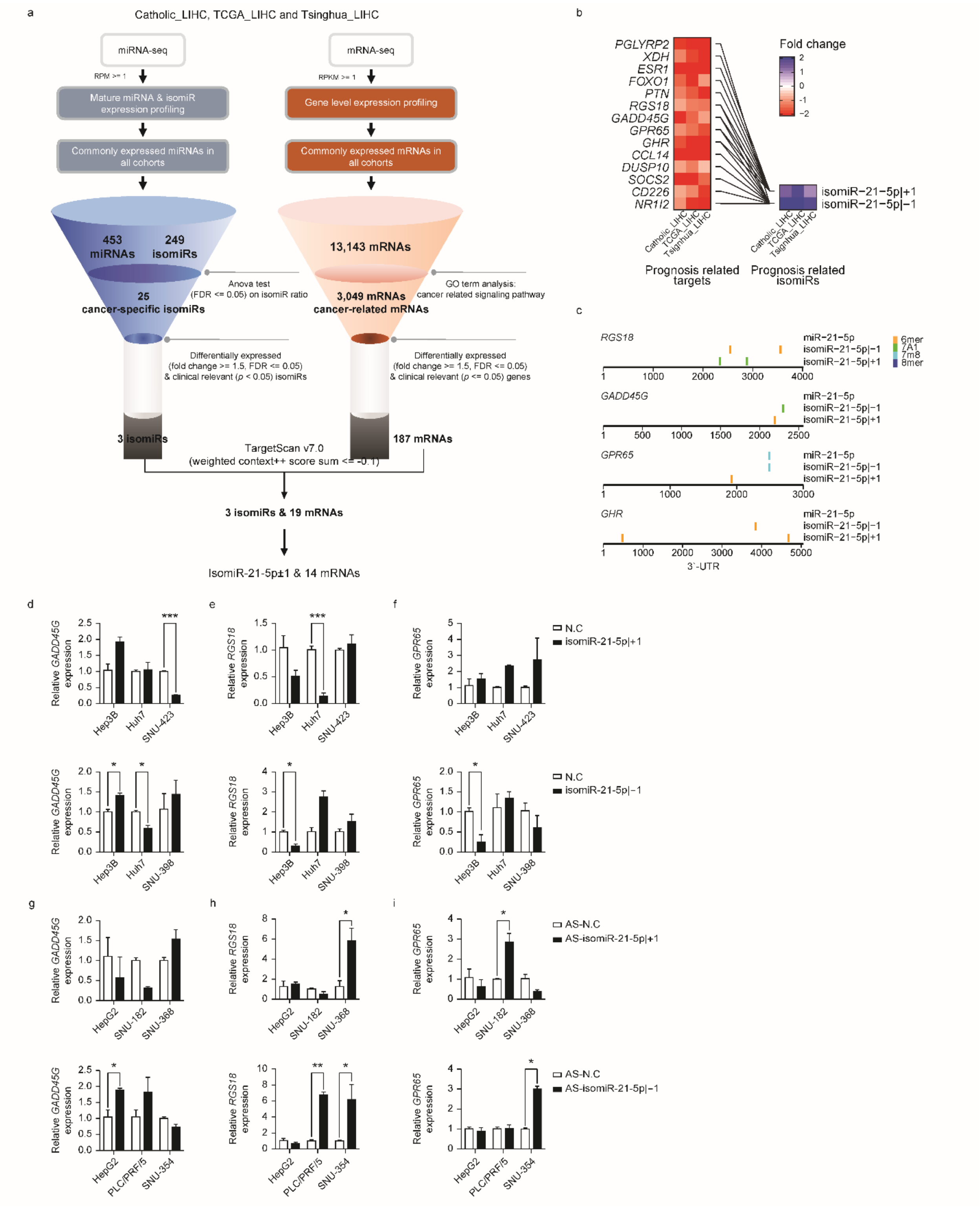

a
Catholic_LIHC, TCGA_LIHC and Tsinghua_LIHC
miRNA-seq
mRNA-seq
Mature miRNA & isomiR expression profiling
Gene level expression profiling
Commonly expressed miRNAs in all cohorts
Commonly expressed mRNAs in all cohorts
453 miRNAs
249 isomiRs
13,143 mRNAs
Anova test (FDR <= 0.05) on isomiR ratio
GO term analysis: cancer related signaling pathway
25 cancer-specific isomiRs
3,049 mRNAs cancer-related mRNAs
Differentially expressed (fold change >= 1.5, FDR <= 0.05) & clinical relevant (p < 0.05) isomiRs
Differentially expressed (fold change >= 1.5, FDR <= 0.05) & clinical relevant (p <= 0.05) genes
3 isomiRs
187 mRNAs
TargetScan v7.0 (weighted context++ score sum <= -0.1)
3 isomiRs & 19 mRNAs
IsomiR-21-5p±1 & 14 mRNAs
b
PGLYRP2
XDH
ESR1
FOXO1
PTN
RGS18
GADD45G
GPR65
GHR
CCL14
DUSP10
SOCS2
CD226
NR1I2
Fold change
isomiR−21−5p|+1
isomiR−21−5p|−1
Catholic_LIHC
TCGA_LIHC
Tsignhua_LIHC
Prognosis related targets
Prognosis related isomiRs
c
RGS18
GADD45G
GPR65
GHR
miR−21−5p
isomiR−21−5p|−1
isomiR−21−5p|+1
6mer
7A1
7m8
8mer
3`-UTR
d
e
f
g
h
i
Relative GADD45G expression
Relative RGS18 expression
Relative GPR65 expression
Hep3B
Huh7
SNU-423
SNU-398
HepG2
SNU-182
SNU-368
PLC/PRF/5
SNU-354
N.C
isomiR-21-5p|+1
isomiR-21-5p|−1
AS-N.C
AS-isomiR-21-5p|+1
AS-isomiR-21-5p|−1

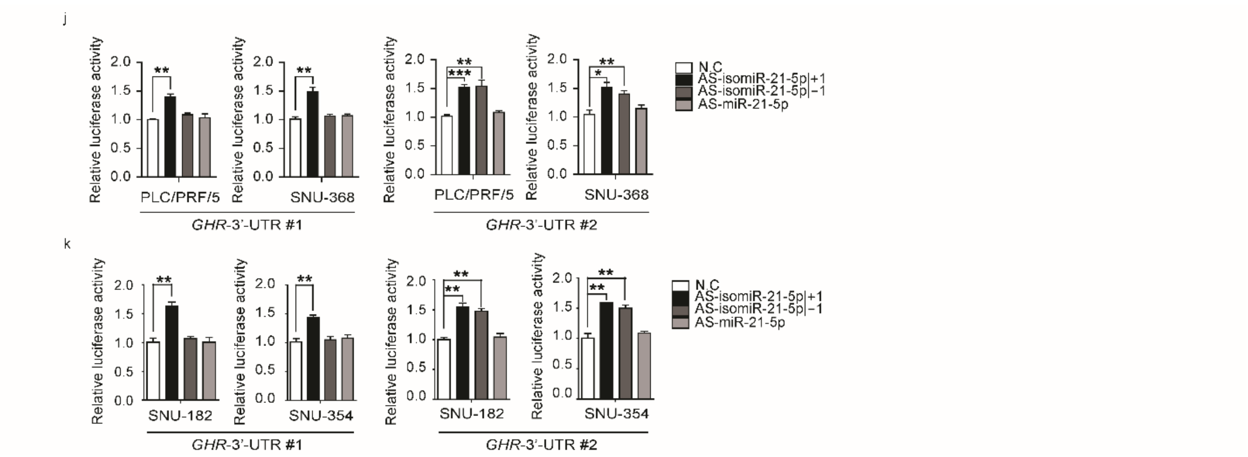

Supplementary Fig. 7 Extended analysis for clinically relevant target of isomiR-21-5p|±1. a Flowchart for the strategy for identifying isomiRs and their targeted genes (see "Prioritization of miRNA/isomiR-target interaction" in the Materials and Methods section for additional details). **b** Heatmap showing the log_2-fold changes, between median expression in non-tumor and HCC samples in each cohort, of the top prioritized isomiR-21-5p|±1 and their targets. **c** Predicted target sites of isomiR-21-5p|±1 in the 3′-UTRs of *RGS18*, *GADD45G*, *GPR65*, and *GHR* with weighted context++ scores ≤ -0.1. **d**–**f** Hep3B, Huh7, and SNU-423 cells were transfected with the isomiR-21-5p|+1 mimic and qRT-PCR was performed to quantify the levels of GADD45G (**d**), RGS18 (**e**), and GPR65 (**f**), upper panel. Hep3B, Huh7, and SNU-398 cells were transfected with the isomiR-21-5p|-1 mimic and qRT-PCR was performed to quantify the levels of GADD45G (**d**), RGS18 (**e**), and GPR65 (**f**), lower panel. **g**–**i** HepG2, SNU-182, and SNU-368 cells were transfected with AS-isomiR-21-5p|+1 and qRT-PCR was performed to quantify the levels of GADD45G (**g**), RGS18 (**h**), and GPR65 (**i**), upper panel. HepG2, PLC/PRF/5, and SNU-354 cells were transfected with AS-isomiR-21-5p|-1 and qRT-PCR was performed to quantify the levels of GADD45G (**g**), RGS18 (**h**), and GPR65 (**i**), lower panel. **j**, **k** PLC/PRF/5 and SNU-354 (isomiR-21-5p|-1-overexpressing cells, **j**) and SNU-182 and SNU-368 (isomiR-21-5p|+1-overexpressing cells, **k**) cells were co-transfected with AS-isomiR-21-5p|+1, AS-isomiR-21-5p|-1, AS-miR-21-5p, or control, and one of two psiCHECK-2-GHR-3′-UTR vectors (*GHR* 3′-UTR Renilla luciferase reporters that also encode firefly luciferase). Both Renilla and firefly luciferase activities are measured in the same sample. Renilla luciferase signals were normalized to firefly luciferase levels. Data in **d**–**k** are represented as mean ± SEM; unpaired Student t-test, *: $P \le 0.05$, **: $P \le 0.01$, and ***: $P \le 0.001$.

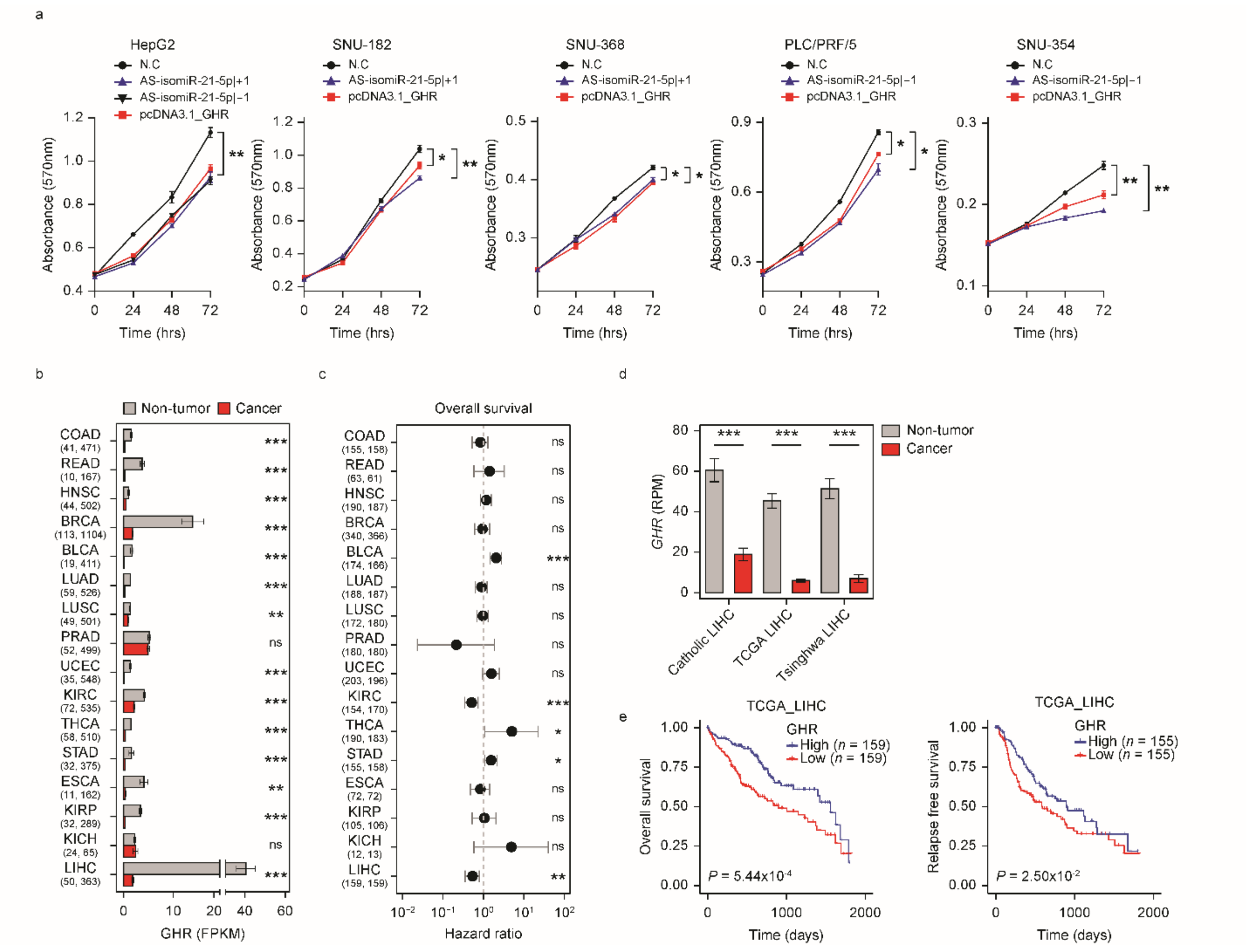

Supplementary Fig. 8 Analyses on GHR function in liver cancer. a HepG2, SNU-182, SNU-368, PLC/PRF/5, and SNU-354 cells were transfected with AS-isomiR-21-5p|+1, AS-isomiR-21-5p|-1, or pcDNA3.1_GHR, and MTT assays were performed to determine the effect on cell proliferation. **b** Comparisons of *GHR* expression levels between non-tumor and cancer samples in different types of cancer in TCGA. The numbers of samples in each case are indicated in parentheses. **c** Forest plots of hazard ratios by cancer type (with 95% confidence intervals) for overall survival, with significant associations with *GHR* expression levels indicated. **d** Comparison of *GHR* expression levels between non-tumor and HCC samples in each dataset. **e** Kaplan-Meier plots for TCGA LIHC patients, grouped based on the median *GHR* expression level, showing overall survival (left) and relapse free survival (right). Data represent the mean ± SEM in **a** the mean ± SD in **b** and **d**; unpaired Student t-test **a** or one-tailed Wilcoxon rank-sum test or one tailed Wilcoxon signed rank-sum test **b** and **d** or Wald test **e**, *: $P \leq 0.05$, **: $P \leq 0.01$, and ***: $P \leq 0.001$.

Supplementary Tables

Supplementary Table 1 miRNA-seq mapping results.

A. Mapping result of Catholic_LIHC miRNA-seq data

Sample	Total Sequenced Reads	Total Mapped Reads	% Mapped Reads
Sample_01	7,373,810	6,443,116	87.38
Sample_02	8,314,358	7,253,531	87.24
Sample_03	8,246,152	6,982,493	84.68
Sample_04	10,638,462	9,826,329	92.37
Sample_05	8,073,848	7,297,508	90.38
Sample_06	6,262,566	5,563,161	88.83
Sample_07	8,168,416	5,578,333	68.29
Sample_08	6,052,408	5,225,901	86.34
Sample_09	5,443,018	4,744,973	87.18
Sample_10	6,974,798	6,024,307	86.37
Sample_11	5,349,856	4,577,688	85.57
Sample_12	6,463,412	5,607,025	86.75
Sample_13	5,839,136	4,574,967	78.35
Sample_14	5,488,366	4,875,554	88.83
Sample_15	4,914,921	4,400,672	89.54
Sample_16	6,170,996	4,815,519	78.03
Sample_17	5,596,551	4,342,012	77.58
Sample_18	5,701,317	4,331,809	75.98
Sample_19	6,213,900	4,541,577	73.09
Sample_20	3,999,679	3,462,372	86.57
Sample_21	5,326,653	3,967,673	74.49
Sample_22	7,996,041	6,810,870	85.18
Sample_23	9,543,348	8,320,487	87.19
Sample_24	5,837,796	4,149,429	71.08
Sample_84	6,119,335	5,144,442	84.07
Sample_26	5,473,253	4,016,310	73.38
Sample_27	7,055,905	5,987,044	84.85
Sample_28	2,372,973	2,057,858	86.72
Sample_29	9,625,688	7,294,659	75.78
Sample_30	8,761,324	7,326,750	83.63
Sample_31	7,111,359	6,220,313	87.47
Sample_32	7,077,535	6,148,488	86.87
Sample_33	5,102,803	4,412,905	86.48
Sample_34	9,722,534	8,862,601	91.16
Sample_35	7,091,028	5,918,282	83.46
Sample_36	6,143,959	5,080,473	82.69
Sample_37	2,304,716	1,221,851	53.02
Sample_38	5,775,345	4,372,573	75.71
Sample_39	5,541,267	4,748,840	85.7
Sample_40	5,628,376	4,908,020	87.2
Sample_41	5,361,695	4,498,616	83.9
Sample_42	5,071,817	4,371,168	86.19
Sample_43	5,893,323	5,040,289	85.53
Sample_44	6,792,814	5,548,050	81.68
Sample_45	10,492,904	9,534,801	90.87
Sample_46	5,831,177	4,875,538	83.61
Sample_47	4,629,760	3,860,870	83.39

Sample_48	5,059,126	4,410,244	87.17
Sample_49	7,042,628	5,875,227	83.42
Sample_50	10,744,802	9,345,199	86.97
Sample_51	8,709,362	7,361,581	84.52
Sample_52	7,352,565	6,153,239	83.69
Sample_53	8,119,912	6,216,200	76.56
Sample_54	8,401,247	7,129,104	84.86
Sample_55	5,902,881	5,290,096	89.62
Sample_56	6,958,082	5,749,989	82.64
Sample_57	5,261,527	4,408,008	83.78
Sample_58	7,040,245	6,039,170	85.78
Sample_59	6,652,958	5,609,448	84.32
Sample_60	7,019,126	5,125,037	73.02
Sample_61	4,016,458	2,783,457	69.3
Sample_62	8,289,979	6,868,825	82.86
Sample_63	7,357,734	6,278,386	85.33
Sample_64	7,377,823	6,382,452	86.51
Sample_65	4,391,868	3,434,994	78.21
Sample_66	2,984,555	2,503,060	83.87
Sample_67	5,670,174	4,815,036	84.92
Sample_69	7,206,091	6,249,314	86.72
Sample_70	3,181,840	2,840,086	89.26
Sample_71	7,105,611	6,090,509	85.71
Sample_72	3,667,992	3,117,554	84.99
Sample_73	4,043,932	2,356,476	58.27
Sample_74	4,823,492	3,935,273	81.59
Sample_75	6,516,201	5,554,182	85.24
Sample_76	5,234,814	4,388,049	83.82

B. Mapping result of TCGA_LIHC miRNA-seq data

Sample	Total Sequenced Reads	Total Mapped Reads	% Mapped Reads
TCGA-BC-A10Q-11A	6,615,569	5,013,770	75.79
TCGA-BC-A10R-11A	6,697,565	5,405,800	80.71
TCGA-BC-A10T-11A	11,667,148	9,107,287	78.06
TCGA-BC-A10U-11A	10,219,940	7,063,025	69.11
TCGA-BC-A10W-11A	6,361,221	5,154,024	81.02
TCGA-BC-A10X-11A	7,338,343	5,662,744	77.17
TCGA-BC-A10Y-11A	8,421,584	6,826,891	81.06
TCGA-BC-A10Z-11A	7,780,048	5,510,604	70.83
TCGA-BC-A110-11A	9,525,457	7,449,185	78.2
TCGA-BC-A216-11A	7,683,728	6,213,768	80.87
TCGA-BD-A2L6-11A	7,058,261	6,161,754	87.3
TCGA-BD-A3EP-11A	3,625,446	3,341,382	92.16
TCGA-DD-A113-11A	4,864,995	3,483,574	71.6
TCGA-DD-A114-11A	8,262,488	6,481,973	78.45
TCGA-DD-A116-11A	4,853,166	4,425,684	91.19
TCGA-DD-A118-11A	10,679,251	9,209,936	86.24
TCGA-DD-A119-11A	6,191,354	4,900,999	79.16
TCGA-DD-A11A-11A	6,299,133	4,950,157	78.58
TCGA-DD-A11B-11A	10,193,263	8,077,251	79.24
TCGA-DD-A11C-11A	3,509,129	2,747,838	78.31
TCGA-DD-A11D-11A	5,531,294	4,350,493	78.65

TCGA-DD-A1EB-11A	11,789,194	9,777,239	82.93
TCGA-DD-A1EC-11A	12,271,506	10,422,550	84.93
TCGA-DD-A1EE-11A	8,089,705	6,778,833	83.8
TCGA-DD-A1EG-11A	8,073,512	7,298,687	90.4
TCGA-DD-A1EH-11A	9,526,929	8,060,441	84.61
TCGA-DD-A1EI-11A	5,160,032	3,976,676	77.07
TCGA-DD-A1EJ-11A	6,702,019	5,551,018	82.83
TCGA-DD-A1EL-11A	5,062,615	4,218,219	83.32
TCGA-DD-A39V-11A	7,131,382	6,464,457	90.65
TCGA-DD-A39W-11A	7,644,225	6,737,857	88.14
TCGA-DD-A39X-11A	6,975,708	6,235,817	89.39
TCGA-DD-A39Z-11A	7,379,664	6,517,861	88.32
TCGA-DD-A3A1-11A	6,638,761	5,792,483	87.25
TCGA-DD-A3A2-11A	6,069,623	5,109,907	84.19
TCGA-DD-A3A3-11A	9,701,909	8,364,596	86.22
TCGA-DD-A3A4-11A	4,126,818	3,677,739	89.12
TCGA-DD-A3A5-11A	3,676,211	3,367,084	91.59
TCGA-DD-A3A6-11A	4,987,403	4,472,213	89.67
TCGA-DD-A3A8-11A	5,704,291	5,025,723	88.1
TCGA-EP-A12J-11A	10,676,034	8,987,934	84.19
TCGA-EP-A26S-11A	7,994,664	6,550,723	81.94
TCGA-EP-A3RK-11A	7,207,957	6,637,245	92.08
TCGA-ES-A2HT-11A	3,926,221	3,511,621	89.44
TCGA-FV-A23B-11A	9,325,075	8,077,097	86.62
TCGA-FV-A2QR-11A	10,742,750	9,747,304	90.73
TCGA-FV-A3I0-11A	7,991,249	7,089,010	88.71
TCGA-FV-A3I1-11A	6,573,412	5,874,122	89.36
TCGA-FV-A3R2-11A	11,152,239	10,272,316	92.11
TCGA-G3-A3CH-11A	6,728,343	5,928,271	88.11
TCGA-2Y-A9GV-01A	4,068,548	3,785,442	93.04
TCGA-2Y-A9H0-01A	3,133,218	2,841,185	90.68
TCGA-2Y-A9H3-01A	7,569,768	6,877,647	90.86
TCGA-3K-AAZ8-01A	2,002,035	1,893,954	94.6
TCGA-5C-AAPD-01A	7,038,501	6,507,339	92.45
TCGA-BC-A10S-01A	4,551,776	3,705,778	81.41
TCGA-BC-A10T-01A	8,557,341	6,793,580	79.39
TCGA-BC-A110-01A	16,292,927	15,074,357	92.52
TCGA-BC-A69I-01A	3,124,878	2,924,710	93.59
TCGA-CC-5260-01A	14,059,956	13,119,251	93.31
TCGA-CC-5262-01A	9,106,858	7,690,170	84.44
TCGA-CC-5263-01A	3,987,199	3,046,613	76.41
TCGA-CC-A123-01A	5,845,413	4,798,708	82.09
TCGA-CC-A3MB-01A	3,124,974	2,846,435	91.09
TCGA-CC-A7IF-01A	7,112,739	5,860,718	82.4
TCGA-CC-A7IH-01A	3,730,599	3,403,821	91.24
TCGA-CC-A7IL-01A	2,238,152	2,015,826	90.07
TCGA-CC-A8HS-01A	2,840,616	2,686,376	94.57
TCGA-DD-A1ED-01A	4,801,898	4,035,178	84.03
TCGA-DD-A3A2-01A	1,943,213	1,515,547	77.99
TCGA-DD-A4NL-01A	2,841,425	2,462,528	86.67
TCGA-DD-A4NO-01A	6,034,445	5,612,151	93
TCGA-DD-A4NV-01A	3,117,146	2,928,654	93.95
TCGA-DD-A73C-01A	3,209,935	2,915,861	90.84
TCGA-DD-A73D-01A	8,938,569	8,400,301	93.98

TCGA-DD-A73E-01A	3,514,415	3,268,084	92.99
TCGA-DD-A73F-01A	5,193,669	4,575,155	88.09
TCGA-DD-AAE4-01A	5,087,043	4,745,322	93.28
TCGA-DD-AAVP-01A	7,161,726	6,720,356	93.84
TCGA-EP-A12J-01A	6,745,540	6,001,450	88.97
TCGA-FV-A2QR-01A	8,411,092	7,794,053	92.66
TCGA-G3-A5SK-01A	4,044,878	3,711,543	91.76
TCGA-G3-A7M7-01A	4,450,457	3,995,486	89.78
TCGA-G3-A7M8-01A	3,417,923	3,185,928	93.21
TCGA-G3-AAV2-01A	4,852,860	4,635,862	95.53
TCGA-G3-AAV4-01A	5,811,809	5,407,556	93.04
TCGA-K7-A5RF-01A	3,536,984	3,164,070	89.46
TCGA-K7-A5RG-01A	8,937,132	8,025,990	89.8
TCGA-KR-A7K0-01A	3,746,992	3,307,087	88.26
TCGA-KR-A7K2-01A	4,338,681	3,878,389	89.39
TCGA-KR-A7K7-01A	5,307,088	4,782,212	90.11
TCGA-KR-A7K8-01A	7,659,765	7,040,137	91.91
TCGA-MI-A75I-01A	5,093,579	4,668,894	91.66
TCGA-MR-A520-01A	2,542,023	2,244,254	88.29
TCGA-NI-A4U2-01A	3,179,902	2,369,785	74.52
TCGA-UB-AA0V-01A	5,974,792	5,586,579	93.5
TCGA-WX-AA46-01A	3,597,791	3,212,237	89.28
TCGA-XR-A8TE-01A	2,982,211	2,807,339	94.14
TCGA-XR-A8TF-01A	2,109,325	1,967,353	93.27
TCGA-ZP-A9CV-01A	5,992,657	5,683,267	94.84
TCGA-ZP-A9CY-01A	4,513,529	4,242,631	94
TCGA-ZP-A9CZ-01A	4,011,467	3,764,815	93.85
TCGA-ZP-A9D0-01A	3,567,486	3,347,830	93.84
TCGA-ZP-A9D4-01A	8,536,765	8,210,212	96.17
TCGA-ZS-A9CE-01A	4,321,085	4,078,596	94.39
TCGA-2Y-A9GS-01A	7,295,503	6,919,290	94.84
TCGA-2Y-A9GT-01A	3,978,629	3,740,949	94.03
TCGA-2Y-A9GU-01A	8,117,055	7,794,144	96.02
TCGA-2Y-A9GW-01A	8,166,146	7,786,558	95.35
TCGA-2Y-A9GX-01A	5,919,519	5,545,273	93.68
TCGA-2Y-A9GZ-01A	8,252,112	7,764,261	94.09
TCGA-2Y-A9H1-01A	4,282,211	3,953,217	92.32
TCGA-2Y-A9H4-01A	5,214,193	4,865,906	93.32
TCGA-2Y-A9H6-01A	9,364,355	8,824,367	94.23
TCGA-2Y-A9H7-01A	5,796,476	5,448,958	94
TCGA-2Y-A9H8-01A	8,496,312	8,018,531	94.38
TCGA-2Y-A9H9-01A	4,351,462	4,074,895	93.64
TCGA-2Y-A9HA-01A	6,462,204	5,958,289	92.2
TCGA-2Y-A9HB-01A	5,178,887	4,868,889	94.01
TCGA-4R-AA8I-01A	6,319,115	5,897,194	93.32
TCGA-5C-A9VG-01A	12,226,125	11,123,216	90.98
TCGA-5C-A9VH-01A	8,055,241	7,675,618	95.29
TCGA-5R-AA1C-01A	9,424,146	8,686,968	92.18
TCGA-5R-AAAM-01A	6,215,490	5,424,300	87.27
TCGA-BC-A10R-01A	9,247,929	8,285,838	89.6
TCGA-BC-A10U-01A	4,560,025	4,062,371	89.09
TCGA-BC-A10X-01A	7,368,762	6,119,173	83.04
TCGA-BC-A10Z-01A	4,621,369	3,444,501	74.53
TCGA-BC-A112-01A	8,160,701	7,061,895	86.54

TCGA-BC-A216-01A	4,946,091	3,121,315	63.11
TCGA-BC-A3KF-01A	3,712,083	3,353,813	90.35
TCGA-BD-A2L6-01A	4,073,483	3,565,433	87.53
TCGA-BD-A3EP-01A	5,759,330	5,169,544	89.76
TCGA-BD-A3ER-01A	6,954,541	6,259,017	90
TCGA-BW-A5NO-01A	5,614,469	5,232,827	93.2
TCGA-CC-5258-01A	5,368,930	4,367,234	81.34
TCGA-CC-5259-01A	4,708,273	4,175,645	88.69
TCGA-CC-5261-01A	8,687,636	7,334,474	84.42
TCGA-CC-5264-01A	4,753,696	3,899,358	82.03
TCGA-CC-A3MA-01A	12,156,779	11,405,932	93.82
TCGA-CC-A3MC-01A	4,371,300	4,090,022	93.57
TCGA-CC-A5UD-01A	9,296,739	8,927,545	96.03
TCGA-CC-A5UE-01A	5,388,417	5,037,023	93.48
TCGA-CC-A7IE-01A	3,179,864	2,916,893	91.73
TCGA-CC-A7IG-01A	6,214,731	5,782,677	93.05
TCGA-CC-A8HT-01A	1,797,305	1,668,575	92.84
TCGA-CC-A8HV-01A	2,901,534	2,773,610	95.59
TCGA-CC-A9FS-01A	5,836,385	5,451,985	93.41
TCGA-CC-A9FU-01A	3,855,766	3,571,129	92.62
TCGA-CC-A9FV-01A	10,407,996	9,095,434	87.39
TCGA-CC-A9FW-01A	4,324,927	4,088,967	94.54
TCGA-DD-A115-01A	6,843,126	5,512,456	80.55
TCGA-DD-A118-01A	7,978,481	7,062,722	88.52
TCGA-DD-A11B-01A	8,722,810	8,057,663	92.37
TCGA-DD-A11D-01A	6,913,487	5,848,133	84.59
TCGA-DD-A1EA-01A	7,708,561	6,649,006	86.25
TCGA-DD-A1EB-01A	7,519,112	6,105,379	81.2
TCGA-DD-A1EI-01A	6,821,733	6,049,710	88.68
TCGA-DD-A1EJ-01A	4,411,705	3,030,454	68.69
TCGA-DD-A1EK-01A	5,718,387	4,637,270	81.09
TCGA-DD-A39W-01A	8,780,910	7,997,498	91.08
TCGA-DD-A39X-01A	4,285,619	3,688,588	86.07
TCGA-DD-A39Z-01A	7,390,177	6,575,897	88.98
TCGA-DD-A3A1-01A	6,343,625	5,448,412	85.89
TCGA-DD-A3A3-01A	5,634,189	5,142,866	91.28
TCGA-DD-A3A5-01A	7,752,239	7,108,381	91.69
TCGA-DD-A3A6-01A	12,560,786	11,452,006	91.17
TCGA-DD-A3A8-01A	2,954,356	2,761,381	93.47
TCGA-DD-A3A9-01A	7,493,954	6,988,250	93.25
TCGA-DD-A4NB-01A	3,177,021	2,597,538	81.76
TCGA-DD-A4NF-01A	5,003,411	4,459,440	89.13
TCGA-DD-A4NG-01A	4,464,618	3,575,466	80.08
TCGA-DD-A4NI-01A	4,998,796	4,412,457	88.27
TCGA-DD-A4NS-01A	3,447,324	3,181,700	92.29
TCGA-DD-A73A-01A	4,335,911	3,891,761	89.76
TCGA-DD-A73B-01A	5,729,337	5,373,913	93.8
TCGA-DD-AAC9-01A	6,984,638	6,638,848	95.05
TCGA-DD-AACC-01A	5,344,054	4,931,936	92.29
TCGA-DD-AACJ-01A	6,217,981	5,889,723	94.72
TCGA-DD-AACK-01A	6,039,515	5,692,674	94.26
TCGA-DD-AACT-01A	5,367,580	5,071,017	94.47
TCGA-DD-AAD0-01A	7,916,879	7,478,643	94.46
TCGA-DD-AAD2-01A	4,978,304	4,696,294	94.34

TCGA-DD-AAD3-01A	9,877,281	9,318,413	94.34
TCGA-DD-AAD8-01A	6,612,453	6,044,671	91.41
TCGA-DD-AADS-01A	7,883,687	7,026,058	89.12
TCGA-DD-AADY-01A	5,143,001	4,797,256	93.28
TCGA-DD-AAE3-01A	5,939,840	4,907,395	82.62
TCGA-DD-AAE6-01A	5,356,387	4,988,399	93.13
TCGA-DD-AAE7-01A	5,127,544	4,814,601	93.9
TCGA-DD-AAEB-01A	7,228,120	6,369,706	88.12
TCGA-DD-AAEH-01A	7,216,573	6,582,378	91.21
TCGA-DD-AAEI-01A	3,648,844	3,383,559	92.73
TCGA-DD-AAVQ-01A	9,828,528	9,310,279	94.73
TCGA-DD-AAVR-01A	7,824,344	6,739,988	86.14
TCGA-DD-AAVS-01A	5,359,217	5,046,622	94.17
TCGA-DD-AAVU-01A	8,046,941	7,550,554	93.83
TCGA-DD-AAVW-01A	4,792,647	4,448,702	92.82
TCGA-DD-AAVX-01A	5,301,020	4,874,696	91.96
TCGA-DD-AAVY-01A	4,735,671	4,216,701	89.04
TCGA-DD-AAVZ-01A	9,877,114	9,426,241	95.44
TCGA-DD-AAW0-01A	6,801,489	6,419,047	94.38
TCGA-DD-AAW1-01A	6,055,688	5,732,022	94.66
TCGA-DD-AAW2-01A	7,239,381	6,720,979	92.84
TCGA-DD-AAW3-01A	6,767,176	6,373,962	94.19
TCGA-ED-A459-01A	2,940,975	2,626,100	89.29
TCGA-ED-A5KG-01A	9,214,124	8,547,642	92.77
TCGA-ED-A627-01A	4,540,321	4,259,664	93.82
TCGA-ED-A7PZ-01A	3,531,139	3,134,622	88.77
TCGA-ED-A7XO-01A	7,720,915	6,112,356	79.17
TCGA-ED-A82E-01A	13,319,833	12,336,746	92.62
TCGA-ED-A97K-01A	9,174,770	8,859,417	96.56
TCGA-EP-A26S-01A	6,874,970	5,567,776	80.99
TCGA-EP-A2KB-01A	4,337,182	3,952,460	91.13
TCGA-EP-A3JL-01A	8,406,273	7,628,064	90.74
TCGA-EP-A3RK-01A	3,540,591	3,202,722	90.46
TCGA-ES-A2HS-01A	3,069,835	2,822,200	91.93
TCGA-ES-A2HT-01A	3,003,380	2,678,780	89.19
TCGA-FV-A2QQ-01A	2,880,476	2,666,291	92.56
TCGA-FV-A3I0-01A	15,878,295	15,290,677	96.3
TCGA-FV-A3I1-01A	5,685,699	5,278,253	92.83
TCGA-FV-A495-01A	5,770,853	5,303,733	91.91
TCGA-FV-A496-01A	7,582,164	7,130,688	94.05
TCGA-FV-A4ZP-01A	3,649,521	2,393,948	65.6
TCGA-FV-A4ZQ-01A	3,649,304	3,294,654	90.28
TCGA-G3-A25S-01A	5,620,654	4,680,586	83.27
TCGA-G3-A25T-01A	9,788,407	8,672,988	88.6
TCGA-G3-A25V-01A	8,410,325	7,286,836	86.64
TCGA-G3-A25Z-01A	6,606,295	5,832,385	88.29
TCGA-G3-A3CG-01A	6,543,872	5,987,928	91.5
TCGA-G3-A3CH-01A	6,631,477	5,820,284	87.77
TCGA-G3-A3CI-01A	8,155,949	7,517,601	92.17
TCGA-G3-A3CJ-01A	9,529,360	8,589,464	90.14
TCGA-G3-A3CK-01A	6,027,184	5,510,046	91.42
TCGA-G3-A5SI-01A	5,056,051	4,665,874	92.28
TCGA-G3-A5SJ-01A	7,609,331	7,079,666	93.04
TCGA-G3-A5SL-01A	5,280,620	4,899,629	92.79

TCGA-G3-A6UC-01A	5,840,265	5,551,517	95.06
TCGA-G3-A7M5-01A	2,398,472	2,228,808	92.93
TCGA-G3-A7M9-01A	6,397,377	5,102,571	79.76
TCGA-G3-AAUZ-01A	5,194,420	4,840,805	93.19
TCGA-G3-AAV0-01A	6,361,514	6,109,758	96.04
TCGA-G3-AAV3-01A	4,067,485	3,835,623	94.3
TCGA-G3-AAV5-01A	3,819,500	3,583,833	93.83
TCGA-G3-AAV7-01A	5,787,746	4,944,317	85.43
TCGA-GJ-A3OU-01A	12,546,732	11,796,716	94.02
TCGA-GJ-A6C0-01A	2,737,487	2,565,592	93.72
TCGA-GJ-A9DB-01A	5,043,194	4,875,079	96.67
TCGA-HP-A5MZ-01A	5,672,148	5,220,114	92.03
TCGA-K7-A6G5-01A	2,136,228	2,007,793	93.99
TCGA-K7-AAU7-01A	9,892,830	9,530,241	96.33
TCGA-LG-A6GG-01A	2,833,284	2,639,816	93.17
TCGA-LG-A9QC-01A	5,023,690	4,708,903	93.73
TCGA-LG-A9QD-01A	5,403,894	5,168,396	95.64
TCGA-MI-A75E-01A	9,707,585	9,062,101	93.35
TCGA-MI-A75G-01A	7,245,282	6,544,973	90.33
TCGA-O8-A75V-01A	8,004,277	7,519,902	93.95
TCGA-PD-A5DF-01A	5,222,385	4,628,019	88.62
TCGA-QA-A7B7-01A	4,932,445	4,612,331	93.51
TCGA-RC-A6M4-01A	6,074,884	5,427,804	89.35
TCGA-RC-A6M5-01A	8,158,207	7,530,150	92.3
TCGA-RC-A7SB-01A	5,437,852	5,045,116	92.78
TCGA-RC-A7SF-01A	8,967,254	8,377,353	93.42
TCGA-RG-A7D4-01A	4,138,259	3,821,302	92.34
TCGA-T1-A6J8-01A	6,932,153	6,552,206	94.52
TCGA-UB-A7MA-01A	5,142,651	4,903,356	95.35
TCGA-UB-A7ME-01A	6,225,684	5,893,081	94.66
TCGA-UB-A7MF-01A	3,995,299	3,704,120	92.71
TCGA-UB-AA0U-01A	7,334,919	6,978,706	95.14
TCGA-WJ-A86L-01A	3,530,476	3,288,442	93.14
TCGA-WQ-AB4B-01A	5,840,568	5,276,623	90.34
TCGA-WX-AA47-01A	4,950,759	4,416,277	89.2
TCGA-XR-A8TC-01A	2,527,665	2,352,061	93.05
TCGA-XR-A8TG-01A	4,996,001	4,689,398	93.86
TCGA-ZP-A9D1-01A	6,939,089	6,566,829	94.64
TCGA-ZP-A9D2-01A	10,584,921	10,216,506	96.52
TCGA-ZS-A9CD-01A	6,980,070	6,554,945	93.91
TCGA-ZS-A9CF-01A	4,158,263	3,709,160	89.2
TCGA-ZS-A9CG-01A	4,355,550	4,012,001	92.11
TCGA-2V-A95S-01A	4,926,063	4,331,840	87.94
TCGA-2Y-A9GY-01A	4,155,112	3,914,326	94.21
TCGA-2Y-A9H2-01A	7,254,123	6,986,501	96.31
TCGA-2Y-A9H5-01A	9,751,466	9,314,383	95.52
TCGA-5R-AA1D-01A	4,352,289	3,814,750	87.65
TCGA-BC-4072-01B	5,181,882	4,398,690	84.89
TCGA-BC-4073-01B	5,869,166	4,385,214	74.72
TCGA-BC-A10W-01A	6,436,283	5,095,794	79.17
TCGA-BC-A10Y-01A	5,845,935	5,003,323	85.59
TCGA-BC-A217-01A	7,194,145	6,151,438	85.51
TCGA-BC-A3KG-01A	10,079,210	8,911,891	88.42
TCGA-BC-A5W4-01A	5,458,309	5,097,209	93.38

TCGA-BC-A69H-01A	3,118,632	2,912,851	93.4
TCGA-BC-A8YO-01A	5,019,484	4,604,232	91.73
TCGA-BW-A5NP-01A	5,031,637	4,587,972	91.18
TCGA-BW-A5NQ-01A	4,765,869	4,243,999	89.05
TCGA-CC-A1HT-01A	8,326,454	6,998,785	84.05
TCGA-CC-A3M9-01A	6,376,910	5,784,748	90.71
TCGA-CC-A5UC-01A	2,540,940	2,205,099	86.78
TCGA-CC-A7II-01A	3,847,458	3,170,638	82.41
TCGA-CC-A7IJ-01A	6,534,686	6,252,627	95.68
TCGA-CC-A7IK-01A	2,990,201	2,730,753	91.32
TCGA-CC-A8HU-01A	2,658,829	2,474,747	93.08
TCGA-DD-A113-01A	4,865,530	4,036,280	82.96
TCGA-DD-A114-01A	17,315,818	15,909,681	91.88
TCGA-DD-A116-01A	5,525,962	3,998,031	72.35
TCGA-DD-A119-01A	6,545,667	5,712,669	87.27
TCGA-DD-A11A-01A	7,922,805	6,569,662	82.92
TCGA-DD-A11C-01A	3,811,327	3,141,338	82.42
TCGA-DD-A1EC-01A	6,941,212	6,007,319	86.55
TCGA-DD-A1EE-01A	5,733,273	5,034,871	87.82
TCGA-DD-A1EF-01A	7,987,634	6,902,058	86.41
TCGA-DD-A1EG-01A	9,026,158	8,368,022	92.71
TCGA-DD-A1EH-01A	9,404,402	8,809,093	93.67
TCGA-DD-A1EL-01A	3,140,974	2,463,193	78.42
TCGA-DD-A39V-01A	9,519,076	8,991,497	94.46
TCGA-DD-A39Y-01A	6,273,424	5,466,964	87.14
TCGA-DD-A3A4-01A	2,591,861	2,337,376	90.18
TCGA-DD-A3A7-01A	5,246,811	4,897,510	93.34
TCGA-DD-A4NA-01A	5,692,172	5,206,929	91.48
TCGA-DD-A4ND-01A	3,974,661	3,629,128	91.31
TCGA-DD-A4NE-01A	7,232,175	6,772,266	93.64
TCGA-DD-A4NH-01A	8,853,049	7,663,079	86.56
TCGA-DD-A4NN-01A	4,331,028	3,847,961	88.85
TCGA-DD-A4NP-01A	4,651,361	4,258,597	91.56
TCGA-DD-A4NQ-01A	3,464,415	2,421,039	69.88
TCGA-DD-A4NR-01A	1,194,460	1,045,797	87.55
TCGA-DD-A73G-01A	6,000,362	5,615,429	93.58
TCGA-DD-AAC8-01A	6,428,429	5,989,762	93.18
TCGA-DD-AACA-01A	5,241,204	4,765,536	90.92
TCGA-DD-AACB-01A	5,535,258	5,164,572	93.3
TCGA-DD-AACE-01A	5,004,090	4,519,548	90.32
TCGA-DD-AACF-01A	4,980,481	4,536,473	91.09
TCGA-DD-AACH-01A	10,362,220	9,915,409	95.69
TCGA-DD-AACI-01A	7,038,854	6,587,592	93.59
TCGA-DD-AACL-01A	3,686,244	3,185,985	86.43
TCGA-DD-AACN-01A	6,903,993	6,567,102	95.12
TCGA-DD-AACO-01A	4,574,029	4,197,974	91.78
TCGA-DD-AACP-01A	5,766,875	5,152,798	89.35
TCGA-DD-AACQ-01A	7,102,447	6,432,039	90.56
TCGA-DD-AACS-01A	6,855,232	6,405,062	93.43
TCGA-DD-AACU-01A	6,123,172	5,768,755	94.21
TCGA-DD-AACV-01A	4,609,066	4,136,191	89.74
TCGA-DD-AACW-01A	4,520,147	4,202,263	92.97
TCGA-DD-AACX-01A	4,294,735	4,013,051	93.44
TCGA-DD-AACY-01A	5,357,338	4,945,307	92.31

TCGA-DD-AAD5-01A	6,447,874	5,770,442	89.49
TCGA-DD-AAD6-01A	8,072,822	7,179,674	88.94
TCGA-DD-AADA-01A	6,580,504	6,274,576	95.35
TCGA-DD-AADC-01A	6,050,815	5,532,614	91.44
TCGA-DD-AADG-01A	5,192,816	4,832,268	93.06
TCGA-DD-AADI-01A	5,984,560	5,642,009	94.28
TCGA-DD-AADJ-01A	4,586,284	4,309,617	93.97
TCGA-DD-AADK-01A	5,299,018	5,023,510	94.8
TCGA-DD-AADM-01A	6,097,913	5,732,136	94
TCGA-DD-AADO-01A	5,090,372	4,772,447	93.75
TCGA-DD-AADP-01A	6,264,877	5,864,771	93.61
TCGA-DD-AADQ-01A	6,494,945	6,015,282	92.61
TCGA-DD-AADR-01A	3,414,954	3,070,845	89.92
TCGA-DD-AADU-01A	3,633,240	2,908,351	80.05
TCGA-DD-AADV-01A	5,238,400	4,935,737	94.22
TCGA-DD-AADW-01A	8,007,121	7,509,006	93.78
TCGA-DD-AAE1-01A	5,790,576	5,409,692	93.42
TCGA-DD-AAE2-01A	10,569,831	9,990,060	94.51
TCGA-DD-AAE9-01A	4,338,811	3,806,258	87.73
TCGA-DD-AAEA-01A	5,415,200	5,024,398	92.78
TCGA-DD-AAED-01A	5,202,577	4,851,485	93.25
TCGA-DD-AAEG-01A	5,817,624	5,359,350	92.12
TCGA-DD-AAEK-01A	5,910,384	5,503,677	93.12
TCGA-DD-AAVV-01A	10,001,431	9,523,949	95.23
TCGA-ED-A4XI-01A	3,572,210	3,219,490	90.13
TCGA-ED-A66X-01A	5,124,505	4,878,844	95.21
TCGA-ED-A66Y-01A	4,127,793	3,897,124	94.41
TCGA-ED-A7PX-01A	12,461,777	11,037,976	88.57
TCGA-ED-A7PY-01A	3,994,988	3,717,215	93.05
TCGA-ED-A7XP-01A	6,440,740	5,717,575	88.77
TCGA-ED-A8O5-01A	4,487,296	4,195,514	93.5
TCGA-ED-A8O6-01A	3,680,370	3,357,432	91.23
TCGA-EP-A2KA-01A	6,532,516	6,131,635	93.86
TCGA-EP-A2KC-01A	5,730,203	5,277,495	92.1
TCGA-G3-A25U-01A	7,162,494	6,182,801	86.32
TCGA-G3-A25X-01A	13,795,853	12,645,268	91.66
TCGA-G3-A25Y-01A	9,325,148	8,339,198	89.43
TCGA-G3-A5SM-01A	5,829,745	4,814,267	82.58
TCGA-G3-A7M6-01A	4,363,737	4,120,058	94.42
TCGA-G3-AAV1-01A	4,020,201	3,717,437	92.47
TCGA-G3-AAV6-01A	4,926,656	4,557,524	92.51
TCGA-MI-A75C-01A	6,751,532	6,416,799	95.04
TCGA-MR-A8JO-01A	4,365,269	4,011,387	91.89
TCGA-NI-A8LF-01A	2,522,075	2,299,148	91.16
TCGA-RC-A6M3-01A	6,557,690	5,989,016	91.33
TCGA-RC-A6M6-01A	8,885,767	8,226,417	92.58
TCGA-RC-A7S9-01A	3,831,696	3,429,280	89.5
TCGA-RC-A7SH-01A	6,835,327	6,483,231	94.85
TCGA-RC-A7SK-01A	6,438,732	5,917,340	91.9
TCGA-UB-A7MB-01A	3,260,013	3,077,643	94.41
TCGA-UB-A7MC-01A	2,473,584	2,206,372	89.2
TCGA-UB-A7MD-01A	6,988,411	6,375,226	91.23
TCGA-WQ-A9G7-01A	3,051,507	2,715,923	89
TCGA-WX-AA44-01A	6,462,764	6,046,787	93.56

TCGA-XR-A8TD-01A	5,311,223	4,319,288	81.32
TCGA-YA-A8S7-01A	6,437,820	6,134,139	95.28
TCGA-DD-AA3A-01A	5,241,888	4,509,125	86.02
TCGA-DD-AACD-01A	5,188,456	4,798,680	92.49
TCGA-DD-AACG-01A	3,083,388	2,867,683	93
TCGA-DD-AACZ-01A	4,322,198	3,411,489	78.93
TCGA-DD-AAD1-01A	10,102,083	9,523,788	94.28
TCGA-DD-AADB-01A	4,611,698	4,304,328	93.33
TCGA-DD-AADD-01A	3,065,259	2,764,768	90.2
TCGA-DD-AADF-01A	5,993,315	5,535,253	92.36
TCGA-DD-AADL-01A	4,015,609	3,542,904	88.23
TCGA-DD-AADN-01A	4,613,301	4,193,283	90.9
TCGA-DD-AAE0-01A	10,358,229	9,780,158	94.42
TCGA-DD-AAEE-01A	5,179,780	4,052,337	78.23

C. Mapping result of Tsinghua_LIHC miRNA-seq data

Sample	Total Sequenced Reads	Total Mapped Reads	% Mapped Reads
SRR3103222	13,598,944	11,443,633	84.15
SRR3103223	4,589,900	2,022,792	44.07
SRR3103224	7,814,185	6,551,813	83.85
SRR3103225	9,088,186	1,534,182	16.88
SRR3103226	1,515,750	1,080,933	71.31
SRR3103227	8,153,458	2,244,482	27.53
SRR3103228	7,700,301	4,609,438	59.86
SRR3103229	10,090,696	5,033,672	49.88
SRR3103230	7,920,494	2,139,460	27.01
SRR3103231	10,667,727	3,710,549	34.78
SRR3103232	2,358,867	1,700,514	72.09
SRR3103233	7,369,990	5,208,724	70.67
SRR3103234	9,115,044	2,467,269	27.07
SRR3103235	9,178,472	4,180,976	45.55
SRR3103236	8,972,193	3,583,810	39.94
SRR3103237	9,899,395	2,171,900	21.94
SRR3103238	8,261,487	1,211,662	14.67
SRR3103239	7,138,246	5,495,291	76.98
SRR3103240	11,128,151	3,743,327	33.64
SRR3103241	2,756,616	1,522,161	55.22
SRR3103242	4,868,874	3,260,365	66.96
SRR3103243	7,527,088	6,201,409	82.39
SRR3103244	5,015,604	2,125,391	42.38
SRR3103245	13,964,527	11,845,970	84.83
SRR3103246	12,047,122	5,852,524	48.58
SRR3103247	7,278,309	5,525,557	75.92
SRR3103248	7,328,104	2,581,192	35.22
SRR3103249	7,528,165	1,676,323	22.27
SRR3103250	8,053,897	5,148,379	63.92
SRR3103251	9,775,505	8,335,751	85.27
SRR3103252	4,024,117	3,181,097	79.05
SRR3103253	10,679,241	7,344,242	68.77
SRR3103254	11,574,870	5,083,641	43.92
SRR3103255	8,598,037	6,033,527	70.17
SRR3103256	11,773,999	3,041,142	25.83
SRR3103257	9,740,432	6,958,661	71.44

SRR3103258	4,977,460	2,991,389	60.1
SRR3103259	8,317,014	3,587,767	43.14
SRR3103260	6,521,228	2,980,707	45.71
SRR3103261	5,246,754	2,155,095	41.07

Supplementary Table 2 Re-annotation of Drosha cleavage sites based on miRNA-seq support.

pri-miRNA	Strand	Offset (difference from miRbase annotation)	Supported by Catholic_LIHC	Supported by TCGA_LIHC	Supported by Tsinghua_LIHC
mir-145	lower	+2	T	F	T
mir-16-2	lower	-1	T	T	T
mir-935	lower	+1	T	T	T
mir-548p	lower	+3	T	T	T
mir-183	lower	+1	T	F	T
mir-425	lower	-1	T	T	T
mir-330	lower	+1	T	F	T
mir-1304	upper	-3	T	T	F
mir-577	upper	-1	T	F	T
mir-652	upper	-1	T	F	T
mir-153-2	upper	-1	T	T	T
mir-324	lower	-2	T	T	T
mir-146b	lower	+1	T	T	F
mir-101-1	upper	-1	T	T	T
mir-181c	lower	+1	T	T	T
mir-24-1	upper	-1	T	T	F
mir-24-2	upper	-1	T	T	F
mir-148b	upper	-1	T	T	T

Supplementary Table 3. qRT-PCR primer sequences used in this study,

Primer		Nucleotide sequence
hsa-miR-21-5p	Forward	5’-TAGCTTATCAGACTGATGTTGA-3’
	Reverse	5’-GTGCAGGGTCCGAGGT-3’
hsa-isomiR-21-5pl+1	Forward	5’-AGCTTATCAGACTGATGTTGACT-3’
	Reverse	5’-GTGCAGGGTCCGAGGT-3’
hsa-isomiR-21-5pl-1	Forward	5’-GTAGCTTATCAGACTGATGTTGA-3’
	Reverse	5’-GTGCAGGGTCCGAGGT-3’
mmu-isomiR-21a-5pl+1	Forward	5’-AGCTTATCAGACTGATGTTGAC-3’
	Reverse	5’-GTGCAGGGTCCGAGGT-3’
mmu-isomiR-21a-5pl-1	Forward	5’-ATAGCTTATCAGACTGATGTTG-3’
	Reverse	5’-GTGCAGGGTCCGAGGT-3’
U6 snRNA	Forward	5’-GCGCGTCGTGAAGCGTTC-3’
	Reverse	5’-GTGCAGGGTCCGAGGT-3’
	RT-Oligo	5’-GTCGTATCCAGTGCAGGGTCCGAGGTAT TCGCACTGGATACGACAAAA ATATG-3’
GHR	Forward	5’-CAAAGAACCTAGGACCCATACAG-3’
	Reverse	5’-CAGATGGAGGTAAACGATGAATTAAAG-3’
GADD45G	Forward	5’-TAACAAGCCAAATCCGAACC-3’
	Reverse	5’-TAGGGGGACCCCAAGAATAC-3’
RGS18	Forward	5’-AGACTGATGCCCCAAAAGAG-3’

	Reverse	5’-ACTGTCTTGTTCCATGAGCTG-3’
GPR65	Forward	5’-TCACCATCCTGATCTGCAAC-3’
	Reverse	5’-TTTTCCTTGTTTTCCGTGGC-3’
hsa-miR-495-3p	Forward	5’-AAACAAACATGGTGCACTTCTT-3’
	Reverse	5’-GTGCAGGGTCCGAGGT-3’
GAPDH	Forward	5’-ACCAGGTGGTCTCCTCTGAC-3’
	Reverse	5’-TGCTGTAGCCAAATTCGTTG-3’

Supplementary Table 4. siRNA or antagomir sequences used in this study.

siRNA or antagomir	Strand	Nucleotide sequence
Negative control	Sense	5'-CCUACGCCACCAAUUUCGU-3'
	Antisense	5'-ACGAAAUUGGUGGCGUAGG-3'
has-miR-21-5p	Sense	5’-UAGCUUAUCAGACUGAUGUUGA-3’
	Antisense	5’-UCAACAUCAGUCUGAUAAGCUA-3’
AS-isomiR-21-5pl+1	Sense	5’-GUCAACAUCAGUCUGAUAAGCU-3’
AS-isomiR-21-5pl-1	Sense	5’-CAACAUCAGUCUGAUAAGCUAC-3’
siGHR	Sense	5’-CAGAUCUCUUAUGCCUUGA-3’
	Antisense	5’-UCAAGGCAUAAGAGAUCUG-3’
sihnRNPC	Sense	5’-GCCUUCGUUCAGUAUGUUAAU-3’
	Antisense	5’-AUUAACAUACUGAACGAAGGC-3’
siU2AF2	Sense	5’-GCACGGUGGACUGAUUCG-3’
	Antisense	5’-CGAAUCAGACCACCGUGC-3’

References

1 Kim, Y. K., Kim, B. & Kim, V. N. Re-evaluation of the roles of DROSHA, Export in 5, and DICER in microRNA biogenesis. *Proc. Natl. Acad. Sci. U. S. A.* **113**, E1881-1889 (2016).

2 Lorenz, R. *et al.* ViennaRNA Package 2.0. *Algorithms Mol. Biol.* **6**, 26 (2011).

3 DePristo, M. A. *et al.* A framework for variation discovery and genotyping using next-generation DNA sequencing data. *Nat. Genet.* **43**, 491-498 (2011).

4 Van der Auwera, G. A. *et al.* From FastQ data to high confidence variant calls: the Genome Analysis Toolkit best practices pipeline. *Curr. Protoc. Bioinformatics* **43**, 11 10 11-33 (2013).

5 Alon, S., Erew, M. & Eisenberg, E. DREAM: a webserver for the identification of editing sites in mature miRNAs using deep sequencing data. *Bioinformatics* **31**, 2568-2570 (2015).

6 Zhang, Q. Analysis of RNA Editing Sites from RNA-Seq Data Using GIREMI. *Methods Mol. Biol.* **1751**, 101-108 (2018).

7 Wang, A. G. *et al.* Gender-dependent hepatic alterations in H-ras12V transgenic mice. *J. Hepatol.* **43**, 836-844 (2005).
